# Systematic review of international clinical guidelines for the promotion of physical activity for the primary prevention of cardiovascular diseases

**DOI:** 10.1186/s12875-021-01409-9

**Published:** 2021-05-19

**Authors:** N. Aerts, D. Le Goff, M. Odorico, J. Y. Le Reste, P. Van Bogaert, L. Peremans, G. Musinguzi, P. Van Royen, H. Bastiaens

**Affiliations:** 1 Department of Primary and Interdisciplinary Care, Faculty of Medicine and Health Sciences, University of Antwerp, Antwerp, Belgium; 2 Department of General Medicine, SPURBO, Université de Bretagne Occidentale, University of West Brittany, 7479 Brest, EA France; 3 Department of Nursing and Midwifery, Faculty of Medicine and Health Sciences, University of Antwerp, Antwerpen, Belgium; 4 Department of Disease Control and Environmental Health, School of Public Health, College of Health Sciences, Makerere University, Kampala, Uganda

**Keywords:** Systematic review, Clinical practice guidelines, Cardiovascular disease, Primary prevention, Behavior change, Lifestyle advice, Cardiovascular risk reduction

## Abstract

**Background:**

Cardiovascular diseases are the world’s leading cause of morbidity and mortality. An active lifestyle is one of the cornerstones in the primary prevention of cardiovascular disease. An initial step in guiding primary prevention programs is to refer to clinical guidelines. We aimed to systematically review clinical practice guidelines on primary prevention of cardiovascular disease and their recommendations regarding physical activity.

**Methods:**

We systematically searched Trip Medical Database, PubMed and Guidelines International Network from January 2012 up to December 2020 using the following search strings: ‘cardiovascular disease’, ‘prevention’, combined with specific cardiovascular disease risk factors. The identified records were screened for relevance and content. We methodologically assessed the selected guidelines using the AGREE II tool. Recommendations were summarized using a consensus-developed extraction form.

**Results:**

After screening, 27 clinical practice guidelines were included, all of which were developed in Western countries and showed consistent rigor of development. Guidelines were consistent about the benefit of regular, moderate-intensity, aerobic physical activity. However, recommendations on strategies to achieve and sustain behavior change varied. Multicomponent interventions, comprising education, counseling and self-management support, are recommended to be delivered by various providers in primary health care or community settings. Guidelines advise to embed patient-centered care and behavioral change techniques in prevention programs.

**Conclusions:**

Current clinical practice guidelines recommend similar PA lifestyle advice and propose various delivery models to be considered in the design of such interventions. Guidelines identify a gap in evidence on the implementation of these recommendations into practice.

**Supplementary Information:**

The online version contains supplementary material available at 10.1186/s12875-021-01409-9.

## Background

Cardiovascular diseases (CVDs) are the number one cause of death worldwide; more people die annually from CVDs than from any other cause. In 2016 alone, an estimated 17.9 million people died from CVDs, accounting for 31% of global mortality. According to estimates of the World Health Organization, nearly 75% of vascular events may be prevented when a combination of cost-effective population-wide and individual interventions are implemented appropriately [1]. Addressing modifiable CVD risk factors can prevent disability and death, and improve quality of life. The most important behavioral risk factors of heart disease and stroke are physical inactivity, unhealthy diet, tobacco use and harmful use of alcohol [2, 3].

Current literature demonstrates numerous methods to reduce CVD risk profile with strong consensus regarding lifestyle behavior. Primary prevention is an important priority for all developers of health policy [4]. Physical activity (PA) is one of the main targeted areas in CVD primary prevention, nested within a broader lifestyle approach and besides medical treatment [5]. Although countries are facing an overall pandemic of physical inactivity similar to that of smoking, the response to the public health challenge of inactivity has not been as strong as needed [6]. Worldwide, one in four adults and three in four adolescents currently do not meet the global recommendations for PA set by the World Health Organization. In some countries, levels of attainment of PA guidelines can be as low as 30% and inactivity accounts for 1–3% of national health care costs [7]. Evidence shows that adults stand to gain substantial longevity benefits by becoming more physically active, irrespective of established CVD risk factors. Increasing and maintaining PA levels to meet the minimum public health recommendations can prevent nearly one in two deaths associated with physical inactivity [8].

Despite high evidence on the importance of lifestyle behavior change interventions, implementation in practice remains limited [9]. Horizon 2020 project SPICES^1^ aims to implement a program, containing PA behavior interventions, for the primary prevention of CVD in primary health care and community settings in various high (Belgium, France, United Kingdom), middle (South Africa) and low (Uganda) income contexts. As improving the efficiency of disseminating the evidence-based practices to practitioners is often seen as a solution for bridging the science‐to‐practice gap [10], a first step for us was to explore the guidelines in order to inform the SPICES program, before evaluating further implementation thereof. Clinical practice guidelines (CPGs) organize and provide the best available evidence to support clinical decision making [11]. Systematically reviewing existing CPGs is an approach that has been used before, however, to our knowledge, no recent study exists that systematically reviewed international CPGs with a focus on PA in the primary prevention of CVD. Our aim is to review guidelines in order to identify best practice recommendations in terms of the design and implementation of interventions, e.g. setting; intervention deliverers; intervention content, for the implementation and evaluation in the Horizon 2020 project SPICES sites.

This systematic review aims to answer the following research question: What recommendations are made in CPGs to guide the design and the implementation of PA interventions in primary health care and at community level, for the primary prevention of CVD?

## Methods 

We applied standard systematic review methodology as outlined by the Cochrane Collaboration [12] and we used the PRISMA^2^ checklist [13] (Additional file 1) for self-evaluation of the overall standards and quality requirements for reporting a systematic literature review. All authors contributed to the development of the research protocol prior to the study.

Between September 2017 and January 2018, NA, PVR and HB carried out a systematic search on Trip Medical Database and International Guidelines Library of the Guidelines International Network (G-I-N) to reach a broad scope of CPGs. An additional systematic search was subsequently carried out on G-I-N and PubMed in December 2020, with the aim of updating the results of this review with the most recently published guidelines. Suitable search strategies were developed for each database, using multiple combinations of free text, MeSH terms, word variants, Boolean operators and truncation for: ‘cardiovascular disease’, ‘prevention’, ‘risk’, ‘lifestyle’, ‘physical activity’. Publication type was restricted to ‘guidelines’, the status was specified to be published or under review and language was restricted to English, Dutch and French.

All records were submitted to a selection procedure on relevance and content, by means of pre-defined in- and exclusion criteria. Publication types other than CPGs and those published before January 2012 were excluded from this review. In case of different versions of the same CPG, we included the most recent one. Titles and abstracts were independently screened by NA, DLG and MO. Records were excluded if both reviewers agreed they were not eligible; discrepancies between reviewers were resolved by discussion until consensus (NA, DLG, MO, JYLR, HB).

CPGs were included if the recommendations described PA interventions for primary prevention of CVD, in comparison to other (lifestyle) intervention or no intervention, targeting the general, adult population. Guidelines needed to report on at least one relevant patient outcome measure commonly used for CVD risk assessment, such as CVD mortality and morbidity, or modifiable risk factors in relation to the primary prevention of CVD (e.g. overweight and obesity, hyperlipidemia, hypertension, lifestyle behavior, dysglycemia). Interventions had to be implemented in primary health care or community settings.

CPGs were excluded if they focused exclusively on CVD risk assessment, pharmacological interventions or lifestyle interventions other than exercise (diet, smoking, alcohol), or if they were explicitly targeting children, adolescents or a geriatric population. Guidelines addressing secondary prevention of CVD, specific conditions related to CVD (e.g. familial hypercholesterolemia, chronic kidney disease, type I diabetes mellitus) and the management of CVD risk factors beyond primary prevention, were excluded from this review as well.

At least two researchers (NA, MO, DLG, JYLR, PVR, PVB, HB) independently performed a quality appraisal of full text records with the AGREE^3^ II instrument. The tool comprises 23 items, organized into six domains: scope and purpose, stakeholder involvement, rigor of development, clarity of presentation, applicability and editorial independence [14]. The reviewers’ overall assessment (scores from 0 to 7, with a consensus-based cut off at a minimum score of 5/7 for inclusion) in combination with a positive advice on use of the guideline (‘yes’ for inclusion), determined the in- or exclusion of each CPG. Records with scores below 5 or around cut-off (one score 4 and one score 5), were excluded. Discrepant scores (more than 1-point difference and one score above 4) and reviewers’ recommendations regarding use of the guideline were discussed until we reached a consensual decision by pooling the data.

In order to ensure accuracy of data extraction for this literature review, an author-team consensus-based data extraction form was determined, comprising of three phases. NA extracted the data, regularly conferring with the senior research team (PVR, LP, PVB, GM, HB). Firstly, we listed all included CPGs and extracted publication year (or year of latest update), country, developing organization, language and title. Secondly, we made an inventory of all PA recommendations, stand alone or as a component of a broader lifestyle recommendation, in order to get an overview of the relevant content of each included CPG with regards to our research questions. If reported, the following characteristics were extracted from each recommendation and its scientific underpinning: strength of recommendation and level of evidence, intervention description and outcomes, implementation strategies, evidence gaps.

Thirdly, two core recommendation matrices (Additional files 2 and 3, to read together with Supplementary material 4 – Grading) were produced: with a listing of relevant recommendations for each CPG; entailing detailed information on clinical relevance and level of evidence grades, primary study intervention characteristics and reported outcomes. Taking into consideration cross-guideline recurrence, results were summarized in Tables 2 and 3.

## Results

**Fig. 1:**
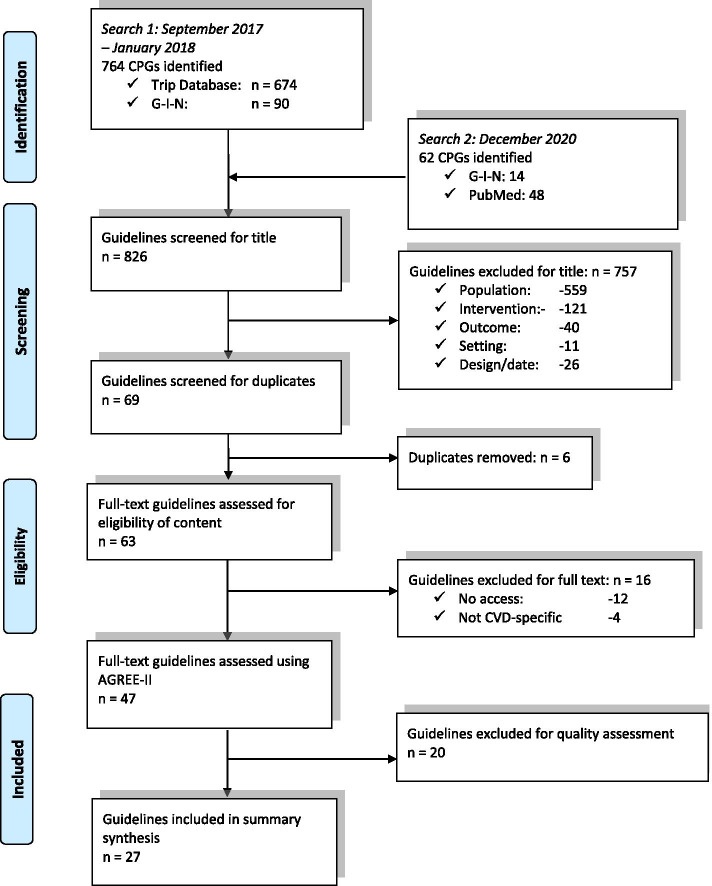
PRISMA flowchart of search, screen and quality assessment process

Our systematic searches retrieved a total of 826 records. After rejection of 757 records based on title and 6 duplicates, 63 CPGs were eligible for full text screening. Finally, 47 CPGs could be withheld, 20 of which did not meet the minimum quality appraisal criteria according to AGREE II. A summary of the full search and review process is presented in a PRISMA flow chart [13] in Fig. 1.

Table 1 summarizes the basic characteristics of the 27 included CPGs, all of which were developed in Western countries. CPGs were categorized according to their main focus. Seven were dedicated entirely to the global prevention of CVD and a further three to lifestyle behavior (LSt), whereas the other CPGs addressed prevention at the level of specific CVD risk factors: seven records on weight management (OW), four on blood lipids (LCh), three on blood pressure control (BP) and three on blood glucose (DM). All included CPGs met the pre-defined minimum quality according to AGREE II criteria. The domain scores showed some variability. Lowest scores were obtained in domain 5 ‘applicability’ (mean 58% [range 25–78%]), highest scores were reached in domain 4 ‘clarity of development’ (median 79% [range 56–94%]). (Additional file5 – AGREE Scores)*.*

**Table 1:** Basic characteristics of included guidelines

CPG code	Year	Country	Developing Organization	Title
*Global cardiovascular disease*				
CVD 1 [15]	2012	Australia	National Vascular Disease Prevention Alliance	Guidelines for the management of absolute cardiovascular disease risk
CVD 2 [16]	2014	UK	National Institute for Health and Care Excellence	Prevention of cardiovascular disease (PH25)
CVD 3 [17]	2016	EU	European Society of Cardiology	European Guidelines on cardiovascular disease prevention in clinical practice
CVD 4 [18]	2017	UK	Scottish Intercollegiate Guidelines Network	Risk estimation and the prevention of cardiovascular disease
CVD 5 [19]	2019	Netherlands	Dutch College of General Practitioners	Cardiovascular risk management (M84)
CVD 6 [20]	2018	Australia	National Heart Foundation of Australia & Cardiac Society of Australia and New Zealand	Guidelines for the prevention, detection, and management of heart failure in Australia
CVD 7 [21]	2019	U.S	American College of Cardiology & American Heart Association Task Force on Clinical Practice Guidelines	Guideline on the primary prevention of cardiovascular disease
*Lifestyle behavior*				
LSt 1 [22]	2012	U.S	U.S. Preventive Services Task Force	Behavioral counseling interventions to promote a healthful diet and physical activity for cardiovascular disease prevention in adults with cardiovascular risk factors
LSt 2 [23]	2014	U.S	American College of Cardiology Foundation & American Heart Association	Guideline on lifestyle management to reduce cardiovascular risk
LSt 3 [24]	2014	UK	National Institute for Health and Care Excellence	Behavior change: individual approaches (PH49)
*Overweight & obesity*				
OW 1 [25]	2012	U.S	U.S. Preventive Services Task Force	Screening for and management of obesity in adults
OW 2 [26]	2013	Australia	National Health and Medical Research Council	Clinical practice guidelines for the management of overweight and obesity in adults, adolescents and children in Australia
OW 3 [27]	2014	U.S	American College of Cardiology Foundation & American Heart Association & The Obesity Society	Guideline for the management of overweight and obesity in adults
OW 4 [28]	2014	U.S	Department of Defense & Department of Veterans Affairs& Veterans Health Administration	Clinical practice guideline for screening and management of overweight and obesity
OW 5 [29]	2014	UK	National Institute for Health and Care Excellence	Obesity prevention (CG43)
OW 6 [30]	2015	Canada	Canadian Task Force on Preventive Health Care	Recommendations for prevention of weight gain and use of behavioral and pharmacological interventions to manage overweight and obesity in adults in primary care
OW 7 [31]	2015	UK	National Institute for Health and Care Excellence	Maintaining a healthy weight and preventing excess weight gain among adults and children
*Blood lipids & cholesterol*				
LCh 1 [32]	2014	UK	National Institute for Health and Care Excellence	Lipid modification: cardiovascular risk assessment and the modification of blood lipids for the primary and secondary prevention of cardiovascular disease (cg181)
LCh 2 [33]	2014	U.S	Department of Defense & Department of Veterans Affairs & Veterans Health Administration	Clinical practice guideline for the management of dyslipidemia for cardiovascular risk reduction
LCh 3 [34]	2018	U.S	American College of Cardiology & American Heart Association Task Force on Clinical Practice Guidelines	Guideline on the management of blood cholesterol
LCh 4 [35]	2019	EU	The Task Force for the management of dyslipidemias of the European Society of Cardiology and European Atherosclerosis Society	Guidelines for the management of dyslipidemias: lipid modification to reduce cardiovascular risk
*Hypertension*				
BP 1 [36]	2014	U.S	Department of Defense & Department of Veterans Affairs & Veterans Health Administration	Clinical practice guideline for the diagnosis and management of hypertension in the primary care setting
BP 2 [37]	2014	U.S	Community Preventive Services Task Force	Team-based care to improve blood pressure control
BP 3 [38]	2020	Canada	Hypertension Canada	Comprehensive guidelines for the prevention, diagnosis, risk assessment, and treatment of hypertension in adults and children
*Blood glucose & type 2 diabetes mellitus*				
DM 1 [39]	2013	Canada	Canadian Diabetes Association	Clinical practice guidelines for the prevention and management of diabetes in Canada: Introduction
DM 2 [40]	2014	UK	National Institute for Health and Care Excellence	Type 2 diabetes prevention: population and community-level interventions (PH35)
DM 3 [41]	2019	EU	European Society of Cardiology & European Association for the Study of Diabetes	Guidelines on diabetes, pre-diabetes, and cardiovascular diseases

The information from the guidelines could be divided into two major categories, including content of PA recommendations and delivery of PA interventions. Table 2 contains all recommendations related to the content of PA interventions; Table 3 contains all recommendations involving the delivery of PA interventions.

**Table 2:** Content of PA interventions for the primary prevention of CVD

Focus of physical activity intervention	Target population	Recommendation	Details of recommendation	Guideline reference number (see Table 1 for details)
Global CVD prevention	General adult population, regardless of CVD risk factors	All adults should be advised to participate in: At least 30 min of moderate intensity (aerobic) PA on at least 5 days of the week (minimum of 150 min/week), or preferably every day of the week	PA: Any bodily movement produced by skeletal muscles that requires energy expenditure Cardiorespiratory fitness: ability of the body to use oxygen to do PA, improved by PA Aerobic PA: movements of large muscle mass in a rhythmic manner for a sustained period Moderate intensity: breathing faster than normal / 3.0–5.9 METS / Increase of breathing rate, heart rate, & warmth, possibly accompanied by sweating / Can be continued for many minutes without exhaustion feeling Prescription of 4 dimensions: Frequency, duration, intensity & type – Taking into account contraindications (individual's condition) Duration: No need for continuous PA to have benefit; longer sessions have no different effect on CHD risk compared with shorter sessions, as long as total energy expenditure is similar	CVD 1 CVD 2 CVD 3 CVD 4 CVD 5 CVD 6 CVD 7 LSt 2 LCh 2 LCh 3 LCh 4 BP 1 BP 3 DM 2
		*OR*		
		At least 15 min of vigorous intensity (aerobic) PA on at least 5 days of the week (minimum of 75 min/week), or preferably every day of the week		CVD 3 CVD 7 LSt 2
		*OR*		
		An equivalent combination thereof, performed in sessions with a duration of at least 10 min/session		CVD 3 CVD 4 CVD 7 OW 4
		PA may include occupational and/or leisure-time activity and should incorporate accumulated bouts of moderate-intensity activities	Type of PA: Active living (non-recreational active travel, household work, gardening), occupational activity (at work), leisure time activity (non-occupational) & exercise (structured and done for specific reason, e.g. brisk walking, cycling, hiking, jogging, swimming)	CVD 4 CVD 5 OW 4 OW 7
		All patients, irrespective of health, fitness or activity level, should be encouraged to increase activity levels gradually Those who are moderately active and are able to increase their activity should be encouraged to do so. Activity can be increased through combination of changes to intensity, duration or frequency For additional benefit in healthy adults, a gradual increase in aerobic PA to 300 min a week of moderate intensity, or 150 min a week of vigorous intensity aerobic PA, or an equivalent combination thereof is recommended	Inverse dose–response relationship between PA levels and CVD risk Potential risk of adverse events associated with vigorous—& high-intensity exercise are extremely low (no significant difference when compared to moderate-intensity PA)	CVD 3 CVD 4 CVD 5 OW 7 LSt 2 LCh 2 DM 3
		Individuals should be advised to minimize the amount of time spent being sedentary (sitting) over extended periods; e.g. by reducing screen time and taking regular breaks from sitting both at home and at work	Provide general advice to minimize periods of prolonged sitting: - High levels of total sedentary behavior are associated with higher risk of CVD & mortality - High levels of sedentary behavior may be associated with additional CVD risk at any level of PA - Undertaking very high levels of PA (> 1 h/day moderate to vigorous PA) may eliminate the association between excess sitting & CVD risk	CVD 3 CVD 4 CVD 5 CVD 7 OW 7
Weight management	Adult population with overweight/ obesity	For adults who are overweight or obese, strongly recommend lifestyle change by participating for ≥ 6 months in comprehensive lifestyle interventions, including: reduced energy intake, increased PA and measures to support behavioral change (behavioral strategies)	Comprehensive lifestyle interventions: multicomponent interventions, with combination of 3 components nutrition, PA & behavior change (BCT). Less amount of activity is needed for weight loss (because of energy deficit from diet + PA together), BCT assists pat in adhering to intervention Prevent weight regain: Maintaining high levels of PA (approximately 60 min per day) combined with other behavioral strategies	CVD 7 LCh 4 OW 2 OW 3 OW 4 OW 7 BP 3
		For adults who are overweight or obese, prescribe approximately 300 min of moderate intensity activity, or 150 min of vigorous activity, or an equivalent combination of moderate intensity and vigorous activities each week combined with reduced dietary intake, to result in weight loss and gradually increase PA levels to prevent weight regain after initial weight loss		CVD 3 OW 2 OW 4
	Adult population with combined CVD risk factors	Counsel overweight and obese adults with CVD risk factors (high BP, hyperlipidemia, hyperglycemia) that lifestyle changes that produce even modest, sustained weight loss of 3–5% produce clinically meaningful health benefits, and greater weight loss produces greater benefits	Dose–response: between amount of weight loss & lowering of BP and improvements in lipid/glycaemia profiles	OW 3
Blood glucose management	Adult population with hyperglycemia or T2DM	A structured program of lifestyle modification that includes moderate weight loss and regular PA should be implemented to reduce the risk of T2DM in individuals with impaired glucose tolerance (prediabetes, IGT) and impaired fasting glucose (IFG) and A1C 6.0–6.4%	Target population for primary prevention: 1. High-risk individuals (e.g. obesity, IGT); 2. High-risk sub-groups (e.g. low SES); 3. General population	CVD 7 DM 1 DM 2 DM 3
	General adult population, adult population with hyperglycemia or T2DM	Advise adults to engage in resistance (muscle-strengthening) training on at least two days a week, such as carrying heavy load, heavy gardening, weight training, push-ups or sit-ups (e.g. 9 exercises, 3 sets & 11 repetitions, intensity 70% of 1-max repetition)	Resistance training: Muscle strengthening of all major muscle groups (legs, hips, back, abdomen, chest, shoulders and arms) Limited evidence for resistance training, but no evidence to exclude it from exercise programs (may confer pat benefits as well) Hypertensive individuals (SBP/DBP of 140–159/90–99 mm Hg): resistance or weight training exercise does not adversely influence the blood pressure T2DM: Specifically for DM prevention, combination of both aerobic & resistance exercise is effective	CVD 3 CVD 4 CVD 5 LSt 2 LCh 1 BP 1 BP 3 DM 3

**Table 3:** Strategies recommended in clinical practice guidelines for the implementation of PA lifestyle advice for the primary prevention of CVD

**Field Subfield**	**Recommendation**	**Details of recommendation**	**Guideline reference number (see Table ****1**** for details)**
**Support & follow-up** *Global CVD prevention—low to medium intensity*	Patient be seen **within one month** of initiation of lifestyle therapy to determine adequacy of risk factor management, degree of patient adherence, presence of adverse effects	Tailor the support and follow-up: Intensity & frequency based on individual need Plan reviews: Before, during & after behavior change intervention to assess progress towards goals Very brief intervention: *(10–15 min)* Target general public & focus on motivation & information Brief intervention: *(15–25 min)* Target low SES people or people whose health/wellbeing could be at risk Extended brief intervention: *(30 min or more)* Target people with high risk behavior; health problems; comorbidities; increased risk of harm; increased need for support to reach/maintain change High intensity intervention: *(over 30 min)* Target people at high risk of causing harm to their health/wellbeing; who have not benefited lower-intensity interventions; who have medical condition that needs specialist advice/monitoring; overweight population who are aiming to lose weight	BP 1
	**Regular assessment and counselling** on PA is recommended to promote the engagement and, if necessary, to support an increase in PA volume over time		CVD 3 CVD 7
	Adults at higher absolute risk of CVD should be given **more frequent and sustained** lifestyle advice, support and follow-up to achieve behavioral change		CVD 1
	Deliver **very brief, brief, extended brief and high intensity** behavior change interventions and programs		LSt 3
	Ensure behavior change is maintained for **at least a year**		LSt 3
	Once the patient's risk CVD factors are controlled, **at least annually follow-up** is suggested (more frequently as indicated), depending on patient preference		BP 1
*Weight management- high intensity*	For **active weight management** in adults, prescribe on-site, high-intensity interventions = ≥ 14 sessions in 6 months with **fortnightly review for the first 3 months, and at least 12 contacts within 12 months).** Assess adherence to the weight loss program by measuring the patient’s weight and providing feedback and ongoing support	Intensive: Multiple contacts over extended periods (5–26 contacts/9–12 months) - *Short-term:* At least weekly - *Intermediate-term:* At least weekly to monthly for another 6 months - *Long-term:* After the first year, at least bimonthly	CVD 7 OW 2 OW 3 OW 4
	Advise overweight and obese patients who have lost weight to participate **long term (≥ 1 year)** in a comprehensive **weight loss maintenance program** consisting of all behavioral components and ongoing support, with additional intervention as required	Continued provision of comprehensive weight loss maintenance program, on-site or by telephone, for periods up to 2,5 years after initial weight loss	CVD 7 OW 3 OW 4
**Behavior change** *Timing*	For adults who are overweight or obese, **discuss readiness to change** lifestyle behaviors	Awareness: Make people aware of their level of CVD risk in relation to lifestyle behavior Timing of the intervention: Conform to current stage of motivation since people are most susceptible for lifestyle change interventions when exposed at a time when they are most open to change (e.g. following profiling results revealing elevated CVD risk)	OW 2
*Counseling content*	Provide **structured information** and combined **behavioral counseling** regarding **lifestyle behaviors** (e.g. healthy diet & PA), in order to prevent CVD and to control CVD risk factors to patients with: 1. normal weight but positive for other CVD risk factors 2. overweight without obesity-associated conditions	Lifestyle: Based on long-standing behavioral patterns, maintained by social environment Content: Focus on behavior change; didactic education & additional support; audit & feedback on progress; strategies for self-monitoring, plan for follow-up Incorporate at least 2 behavior change strategies: Match with patient's needs; other evidence-based effective behavior change techniques; define rationale for techniques included; evaluate novel techniques (limited evidence) Individualized counseling & care plan: patient-centered care as basis for motivation & commitment	OW 4 LSt 1 OW 1 OW 6
	The use of **established (proven) cognitive-behavioral strategies** (e.g. **motivational interviewing**) to facilitate lifestyle change by evoking patient motivation to accept and participate in lifestyle treatments are recommended when designing interventions	Goal setting: Specific, proximal, realistic, personal goals for behavior change/resulting outcomes to achieve/maintaining benefits. Moving forward in small, consecutive steps for changing long-term behavior). Consider achievement of outcomes & review further plans/goals Action planning: Develop & prioritize actions, e.g. PA activity of choice & incorporated in daily life (developing routines & habits) for sustainability & acceptability Problem solving: Well-rehearsed coping plans to prevent/manage relapse, e.g. stimulus control, changes in physical environment Motivational interviewing: Encouraging, enabling, verbal persuasion, modelling exercising behavior, discussing positive effects Other techniques: Self-efficacy (Empower patients by building confidence); Feedback & monitoring (Encourage self-monitoring of behavior/outcomes, provide feedback at regular intervals); Social support (Advise /arrange for social network -family, friends, peers- to provide practical help, emotional support, praise or reward); Cognitive behavioral strategies; Positive reinforcement; Cognitive restructuring; Shared decision-making (between HCP & pat/family)	CVD 3 CVD 5 LSt 3 LCh 4 OW 4
**Provider** *Team-based care*	**Team-based care** with the involvement of **multidisciplinary professionals** is recommended	Multifaceted approach, supporting: Clinical decision-making, collaboration among providers, patient and family member participation Team composition: Trained professionals—dietician/nutritionist, physiotherapist/exercise professional, health educator, psychologist, GP, nurse, pharmacist, social worker, community health worker Roles & responsibility: Limited evidence on organization of complementary competencies Task shifting and sharing: Adding new staff or changing roles of existing staff, considering licensure and responsibilities. E.g. for delivery in primary health care: Brief lifestyle interventions delivered by PN are more cost-effective than delivered by GP Initiation of treatment & follow-up by credentialed provider (e.g. exercise on GP prescription; further educative/follow-up counseling & progress/adherence assessments by other HCP than clinician (e.g. nurse-directed behavioral management) Communication & coordination among various team members	BP 1 BP 2 CVD 3 CVD 5 CVD 7
	Involve **lay or peer workers** to deliver interventions in high risk communities and ensure they are part of a wider team led by health care providers	Involve peers/family in planning, design and delivery of credible appropriate messages and interventions (including helping people to develop practical skills to adopt healthy lifestyle). Management & supervision by professionals	DM 2
	Lay/peer workers & HCP should identify and encourage '**community champions**' (e.g. religious and community leaders) to promote PA	Encourage lay & peer workers to get other members of their community involved	DM 2
*Training*	Provide **training for all professional practitioners and lay people** who are responsible for and/or involved in helping to change people's behavior	Competency & confidence/motivation in: Person-centered care; insight in factors affecting behavior change (incl. psychological, social, cultural & economic) & adverse behaviors; health inequalities; select & tailor appropriate evidence-based interventions; intervention mechanism of action; behavior change techniques; access & refer people to local support services Training model: Focused/structured; based on evidence based content & training models; practice new skills in community/practice, share knowledge amongst peers; identify skills gaps Tailored to: setting, participant's characteristics, focus/priority (integral to main role vs. additional task)	LSt 3 DM 2 BP 1
	**Monitor/assess** behavior change practitioners, **provide feedback** and **give time/support** to develop and maintain competencies	Monitoring & assessment: Competency frameworks & techniques (audio/video recording, observation tool) to monitor HCP’s knowledge & skills (personal development plans, annual reviews), keep up-to-date Ongoing development: Regular evaluation of outcome & process (e.g. using participant feedback), supported by feedback (oral/written), refresher trainings and clear action plans & goal setting in acquiring the necessary competences	LSt 3 DM 2
**Information & education** *Communi-cation*	Provide **patient education** and **clearly communicate** in order to encourage the person to participate in reducing their CVD risk	Health education principles: Small, comprehensive amounts, didactic education and additional support, reinforced by resources (e.g. written, web-based, audiovisual materials) Effective communication: Friendly & positive interaction; non-judgmental interaction (e.g. lower SES groups/minority groups), patient-centered; open-ended questions, reflective listening; show empathy Content: Risk assessment; treatment; impact & benefits of behavior change; being more physically active and improving dietary habits; gradual improvements to PA; interventions/services available & how to use them	OW 7 BP 1 LCh 1
	Exercise **prescription by physicians** (especially GPs), similar to drug prescription, should be considered for health promotion		CVD 3
*Sensibili-zation*	Convey **messages to the local population** and **use community resources** to raise awareness and increase accessibility, such as short term community-based educational programs	Lifestyle messages: consistent, clear, culturally appropriate, integrated within other local health promotion campaigns/interventions Tailor messages to local community: Work with local practitioners, role models & peers; address misconceptions acting as a barrier; disseminate locally to groups at higher risk (e.g. low SES) Channels of delivery: Involve local community (e.g. Community-wide campaigns, social media, local newspapers/radio channels/shops & businesses/events, social establishments, educational institutions, workplaces, places of worship, local health care establishments, community organizations)	CVD 3 DM 2
**Patient-centered care**	**Tailor** interventions for specific groups and individuals in order to ensure interventions meet **individual needs, preferences & circumstances** and are **culturally appropriate** (especially in high-risk communities). **Social determinants** of health should inform optimal implementation of treatment recommendations	Patient participation: At each step, beginning with assessment of ‘readiness to change’ & intention, capability, opportunity & motivation (e.g. if multiple behaviors need to be changed, assess which one the person is most motivated to tackle) Socioeconomic inequalities: determinants for CVD risk. Tailor advice to SES Individualized approach & communication: *Assess & address* previous experiences, beliefs on perceived ability to change, thoughts, worries, attitudes, knowledge, context (physical, economic & social environment), physical and psychological capacity, skills, obstacles, feelings, stage of motivation, skills, self-confidence, barriers to change, self-image, group norms and level of autonomy *& tailor interventions and strategies to meet individual needs*	CVD 7 LSt 3 LCh 4 DM 2 OW 7
	**Shared decision-making** should guide discussions about the best strategies to reduce CVD risk	Decisions should be collaborative between a clinician and a patient: Engage patients in discussions about personalized CVD risk estimates and their implications for the perceived benefits of preventive strategies (i.e. lifestyle habits & goals); hereby addressing potential barriers to treatment options	CVD 7
	Reach a **shared understanding** with overweight and obese patient about the risks of overweight and obesity and the benefits of weight management	1. Ask permission to discuss health risks & potential benefits/risks of interventions 2. Explore understanding, knowledge, beliefs, experience, values, family/social network 3. Share information about potential risks based on health status 4. Emphasize the need for ongoing commitment 5. Provide small amounts of information/advice, tailored to individual values/preferences & easy to understand 6. Use teach-back method to confirm shared understanding	OW 4
**Self- management**	For adults who achieve initial weight loss, strongly recommend the adoption of **specific strategies, appropriate to their individual situation**, to minimize weight regain	Strategies: Self-monitoring (e.g. regular self-weighing), tracking PA (mHealth/eHealth tools or noting activity in diary), relapse prevention & management (rehearsing action-plans e.g. contacting GP), development of routine, coping, self-care strategies	OW 2 CVD 5
	For adults, include a **self-management** and/or **self-monitoring** approach to monitor their weight, BP, or associated behaviors	NOT stand-alone: Self-management approach as part of multicomponent intervention Self-monitoring of chosen behavior or goal (diet/PA/body weight) at least weekly for therapy adherence	OW 2 OW 7 BP 1
	Consider the use of a **self-monitoring device/tracking system** (e.g. pedometer, mobile apps) to increase adherence to PA	Internet-based programs for goal-setting/reminders; lifestyle diaries	BP 1 LCh 1 OW 7
**Setting & referral** *Primary health care*	Managers and health professionals in all **primary care settings** should ensure that preventing and managing obesity is a priority at both strategic and delivery levels. Dedicated resources should be allocated for action	*Brief interventions* in PHC	OW 5
*Community*	Use **community links**, outreach projects and lay or peer workers (from lower SES groups) to deliver interventions	Community-based support: Community health workers assisting HCP & pat by serving as liaisons tot the HC system & lay educators	DM 2
	**Commercial-based programs** that provide a comprehensive lifestyle intervention can be prescribed for weight loss, provided there is peer-reviewed published evidence of safety/efficacy	Community schemes/facilities: Support & promote those that improve access to PA, combined with tailored information based on local needs	OW 3
*Navigation*	Work in **partnership** to develop cost-effective PA interventions	Multifaceted approaches with linkage between PHC—community—public health & health policy interventions	DM 2
	**Provide (written) information** on local, affordable, practical and (culturally) acceptable opportunities for PA		DM 2
	Recognize that people may **need support to change** their lifestyle. To help them do this, refer them to programs such as **exercise referral schemes**	If no in-house program available or cost-effective option	LCh 1
**Delivery mode**	Offer comprehensive lifestyle interventions 1. **face-to-face** in either **individual or group** sessions 2. **telephone based**, either as an alternative or an adjunct to face-to-face intervention, provided it includes personalized feedback from trained practitioner 3. **internet-based**, either as an alternative or an adjunct to face-to-face intervention, provided it includes personalized feedback from trained practitioner	Providing interventions to groups: Group discussions, group tasks (promoting interaction/bonding), mutual support within the group Remote intervention delivery: If there is evidence of efficacy (e.g. telephone, text messaging, apps, internet) for cost-effectiveness	OW 3 OW 4 LSt 1

In the included guidelines, PA for the primary prevention of CVD was described by four dimensions: intensity, duration, frequency and type of the recommended PA activity. All CPGs advised interventions to involve moderate to vigorous PA intensity and a duration of PA sessions of at least 150 min weekly for moderate, or at least 75 min weekly for vigorous intensity PA. Four CPGs reported that several shorter PA sessions were as effective as one session of 30 min daily as they provided a similar total energy expenditure [17, 18, 21, 28]. The CPGs stated that PA should be conducted on a regular basis, meaning on at least five days of the week, preferably each day of the week [16, 20]. Aerobic PA was reported to be the fundamental type of PA for the primary prevention of CVD in eight of the included guidelines [17, 18, 23, 27, 28, 32, 35, 36], which should entail occupational, leisure time, exercise and/or active living activities. Two guidelines recommended interventions with a combination of both aerobic and resistance training for the prevention of diabetes and its CVD complications [17, 41]. Three other guidelines advised on including resistance training or muscle strengthening exercises for the primary prevention of CVD, such as carrying heavy load, heavy gardening, weight training, push-ups or sit-ups on at least two days a week [18, 19, 32], whereas three other CPGs merely stated that there is no evidence for excluding it from interventions [23, 36, 38].

Due to the inverse dose–response relationship between higher levels of PA and lower risk of CVD events as reported in the CPGs [18, 23, 33], a gradual increase of PA levels through a combination of changes to intensity, duration and/or frequency [17–19, 31, 41] should be encouraged. For example, a gradual increase in aerobic PA to 300 min a week of moderate intensity, or 150 min a week of vigorous intensity aerobic PA, or an equivalent combination thereof, is recommended for additional health benefits.

Specifically for the weight management in an adult overweight or obese population, the included guidelines proposed higher-intensity (duration of at least 6 months) comprehensive lifestyle interventions, including high basic levels (and gradual increase) of PA, diet and behavior change components [21, 26–28, 31, 35, 38]. Regular PA with the aim of moderate weight loss is also advised to reduce the risk of Type 2 Diabetes in adults with impaired glucose intolerance and impaired fasting glucose [21, 39, 40]. Five CPGs defined sedentary behavior as an independent CVD risk factor and urged to minimize the amount of time spent being sedentary over extended periods [18, 19, 21], by advising sedentary people to start PA at low intensity and progress gradually [17], and to reduce screen time and take breaks from prolonged sitting both at home and at work [31].

The included CPGs stated that PA interventions designed in line with these recommendations, will result in a decrease in CVD mortality and morbidity. Moreover, a wide range of indirect health benefits were reported in the guidelines, such as: a decrease of systolic and diastolic blood pressure, body fat, body weight, LDL-C, triglycerides, total cholesterol, HbA1c levels and new onset type 2 diabetes mellitus (T2DM); and an increase of HDL-C and insulin sensitivity.

The recommendations on strategies for delivery and implementation of PA made by the included guidelines, could be structured into eight major categories: Support & follow up, Behavior change, Provider, Information & education, Patient-centered care, Self-management, Setting & referral, and Delivery mode.

Behavior change interventions are recommended to be preceded by raising awareness of the individual CVD risk in relation to lifestyle behavior and an assessment of the ‘readiness to change’. It is advised to adapt the timing of such interventions to the stage of motivation, since people are most susceptible for lifestyle change interventions when they are sensitive to change [26]. Guidelines recommended to provide structured counseling targeting lifestyle behaviors [22, 25, 28, 30], incorporating the use of cognitive-behavioral change techniques throughout the multicomponent interventions (e.g. motivational interviewing, shared decision-making, goal-setting, action planning and problem-solving) [17, 19, 24, 28, 35]. Guidelines stated the importance of providing education and communicating clearly with individual patients about all aspects of PA interventions, according to health education principles (e.g. comprehensive amounts of information, reinforced by resources) and using elements of effective communication (e.g. non-judgmental interaction, reflective listening, showing empathy) [31, 32, 35, 36], hereby creating a shared understanding [21, 28]. Two guidelines also advised to convey tailored messages to local populations using community resources, in order to raise general awareness [17, 40]. Patient-centered care was recommended to entail tailoring interventions to groups and individuals and individualizing care plans throughout the entire follow-up pathway, as interventions are advised to meet individual needs, preferences and circumstances, taking into account social determinants of health [21, 24, 31, 35, 40]. CPGs also proposed to integrate follow-up support and self-management strategies, such as self-monitoring [31], PA tracking [31, 32, 36] and relapse management [26] as part of a multicomponent intervention, in order to ensure that initial behavior change is maintained long-term [19, 24, 28, 36]. The use of mHealth or eHealth applications was proposed to support self-management and follow-up interventions [17, 36].

Team-based multidisciplinary care was advised and guidelines recommended various ways of involving both professional and non-professional care providers [17, 19, 21, 29, 37], and linking medical and lay people, peers and family in the planning, design and delivery of interventions [40]. CPGs reported various interprofessional collaboration models with clinicians and non-clinicians, and recommended organizing complementary competencies to be most beneficial for people [17], e.g. by task sharing and shifting in primary health care [36]. We identified several recommendations around community-based support of behavior change interventions through the involvement of community health workers, welfare organizations and social peer support. The included guidelines proposed multifaceted approaches with a clear linkage between primary health care and the community (e.g. by informing people and increase access towards opportunities for increasing PA behavior in the community), in order to increase the effectiveness of interventions [22, 27, 32, 40]. However, they emphasized the importance of embedding lay/peer-led components in a wider team led by health care professionals [40] and underlined the need for appropriate training of both professionals and non-professionals involved in behavior change interventions [24, 36, 40]. CPGs reported various advice regarding intensity and frequency of support and follow-up interventions, emphasizing that it is crucial to tailor this to the needs of each individual. They differentiated between very brief, brief or extended brief interventions, and recommended follow-up for at least one year [15, 17, 24, 36]. High intensity interventions, with multiple contacts over extended periods, were recommended for active weight management and maintenance in three guidelines [26–28]. The guidelines reported no clear precedence in group versus individual and face to face versus additional remote contacts (e.g. telephone or web-based) [22, 27].

The particular intervention delivery strategies as recommended in the included guidelines, can lead to improvement of the following non-clinical outcomes: Increase of motivation and self-efficacy, better adherence to behavioral elements of the interventions, higher participation and attendance rates in treatment activities, better coping with illness, and higher self-reported health behavior.

## Discussion

The objective of this systematic review of guidelines was to identify recommendations regarding the design and implementation of PA interventions for the primary prevention of CVD on primary health care and community level supporting clinical practice, for the implementation and evaluation in the Horizon 2020 project SPICES sites. Using a systematic and comprehensive approach, we selected 27 high quality CPGs and summarized recommendations on the content of PA advice and provided an overview of recommended strategies for the delivery of PA interventions. The strength of this systematic review and pragmatic summary is that it can guide practitioners in designing and implementing PA interventions, embedded in a broader lifestyle program.

All CPGs alluded to a healthy lifestyle including regular PA as representing a major component of primary CVD prevention and should be recommended to the whole population. These findings are in line with more recent systematic reviews of primary studies, which concluded that given the great health benefits, comprehensively tackling multiple lifestyle risk factors should be the cornerstone for reducing the global disease burden [42, 43]. Overall, the content (frequency, duration, intensity) of the PA message that should be given to the adult population in order to lower their CVD risk, was consistently outlined throughout the included CPGs. In their systematic review, Kraus and colleagues studied the different relationships between PA levels and patient outcomes and found that the associations of PA with beneficial health outcomes begin when adopting even very modest levels; meeting the recommendations reduces mortality and CVD risk to about 75 percent of the maximal benefit obtained by PA alone; and PA levels beyond the guidelines’ recommended levels reduce risk even more [44]. Nevertheless, the included CPGs identified an important gap in evidence regarding long-term effectiveness (interventions follow-up beyond 2–3 years), effect on CVD morbidity and mortality and the minimum required PA levels required to gain health benefits. We also found that recommendations remained inconclusive regarding advice on resistance training. Some CPGs suggested that a combination of aerobic PA and resistance training could be effective for people with T2DM, yet limited evidence on effectiveness in CVD protection was reported. Primary studies examining combined resistance and aerobic training reported that taking on both forms of exercise was effective for preventing and managing CVD [45, 46], and it was associated with decreases in body weight, BMI and abdominal subcutaneous fat, and improvements to abdominal fat, visceral fat, cardio-respiratory fitness and HbA1c levels [47]. However, a recent systematic review found insufficient evidence to determine the potential beneficial effect of resistance training on non-fatal events or the effect of substituting aerobic exercise with resistance training [48]. Five of the included CPGs in our review defined sedentary behavior as an independent risk factor for CVD morbidity and mortality. This is in line with a systematic review which concluded that higher levels of total daily sitting time are associated with an increased risk of CVD and diabetes, independent of PA. The authors suggested that reductions in total daily sitting should be recommended in public health guidelines [45]. However, there is insufficient evidence to support the assumption that decreasing sitting time would be easier than effectively promoting PA and evidence remains unclear whether reductions in sedentary time are associated with improvements to the CVD risk [49]. Moreover, given the relative independence of sedentary behavior from PA, it is unsure if existing evidence-based behavioral strategies for increasing PA will also directly decrease sedentary behavior [50].

Various strategies for the delivery of interventions to achieve and sustain behavioral change for healthy lifestyle behaviors such as PA, were recommended by the included guidelines. Most CPGs recommended the use of multicomponent interventions, however, they remained unclear as to which are the most essential components in a package of interventions. Indeed, complex interventions make it difficult to define what exactly are the ‘active ingredients’ of an intervention and how they relate to each other, due to various interacting components; target behavior(s), groups or organizational levels targeted by the intervention; variability of outcomes; and the need for tailoring of the intervention [51]. The use of behavior change techniques was strongly recommended in the included CPGs, although the developers identified an urgent need for future research to examine the most effective approach to deal with multiple behaviors; and the effectiveness of individual techniques on motivation and adherence. Recent studies showed that a combination of education and cognitive-behavioral strategies appears to be more effective than a single intervention [52]. Interventions incorporating cognitive behavioral strategies, including goal-setting, action planning, self-monitoring, feedback and reinforcement are more likely to induce changes [53], as does increasing self-efficacy and action control skills [54]. In some of the guidelines, technology was recommended as opportunity to improve provider-patient communication, self-monitoring, and patient motivation. Current literature reports a disconnect between behavioral strategies shown to be efficacious in face-to-face studies and the implementation of these strategies in technology-delivered interventions. The most common types of strategies (feedback, self-monitoring, and goal setting) are often integrated in technology interventions, whereas other evidence-based treatment components, e.g. barriers identification, relapse prevention, role modeling, motivational interviewing, are not [50]. Team-based care, involving multidisciplinary professionals, was recommended in the included guidelines. They proposed task shifting and sharing strategies to meet time and resource limitations of primary care staff and in addition, engaging other deliverers in the community. In a systematic review of Fisher et al., peer support was shown to have effects in encouraging and helping to sustain a variety of complex health behaviors in prevention and disease management and in areas such as cardiovascular disease, HIV/AIDS, diabetes and other chronic diseases [55]. Optimizing the engagement of innovative providers requires clear definition of roles and scopes of practice, in-service training and formal supervision, and sensitization of health managers to the importance of counselling [56].

The included CPGs also identified some gaps in research and practice implications. The design and implementation of PA lifestyle interventions do bring resource implications, and guidelines proposed that future research should focus on the most effective and cost-effective ways of developing, implementing and assessing tailored and culturally appropriate interventions on primary care and community level. According to the CPGs, delivery of lifestyle advice requires a rigorous analysis of and tailoring to the context, vulnerable target population and individual. The guidelines reported that there was insufficient evidence available to give specific advice on particular population groups such as ethnic minority groups or different socioeconomic groups; yet they emphasized the importance of identifying and managing the needs of different population groups to address inequalities in health. CPGs could not report consistent information on acceptability and adherence to changes in different population groups, interactions between behaviors and processes for change and (cost-) effectiveness of interventions and strategies for those at higher risk or the entire population. Moreover, the guidelines identified a gap in evidence regarding factors that can influence implementation of the recommendations into practice.

This review has some limitations. All included CPGs in this review were developed in high-income western countries with extensive resources, whereas low-and middle-income countries might require a different approach. Second, our used strategy and instruments did not include an analysis of the CPGs’ consistency, meaning that we did not evaluate the underlying strategies of summary and interpretation of the scientific evidence as well as the interpretation and formulation of the recommendations, leading to a possible interpretation bias.

By bringing the advice of current CPGs together in this review, we provided a comprehensive overview of reported evidence-based recommendations for stakeholders that are involved in the design and implementation of PA interventions in primary prevention programs. However, we acknowledge that additional steps are necessary to actually change practice and policy. Implementation studies, such as the SPICES project, can give more insight into contextual barriers and facilitators from the evaluation of implementation outcomes and process, so that closing the chasm between research and practice can be supported.

## Conclusions

Current high-quality CPGs consistently highlight the importance of lifestyle interventions in primary prevention programs for CVD, with PA as one of the major components. PA interventions should be actively integrated in primary health care and community settings. Current clinical practice guidelines recommend similar PA lifestyle advice, and they propose various delivery models to be considered in the design of such interventions. Guidelines identify a gap in evidence on the contextual barriers and facilitators to implementation of these recommendations, urging for future research to focus on closing the gap between research and practice.

## Supplementary Information

**Additional file 1.**

**Additional file 2.**

**Additional file 3.**

**Additional file 4.**

**Additional file 5.**

## Data Availability

All data generated or analyzed during this study are included in this published article and its supplementary information files.

## Abbreviations

CVD: Cardiovascular disease
PA: Physical activity
CPG: Clinical practice guideline
FR: France
ZA: South Africa
UG: Uganda
LSt: Lifestyle behavior guideline
OW: Weight management guideline
LCh: Blood lipids guideline
BP: Blood pressure control guideline
DM: Blood glucose guideline
T2DM: Type 2 diabetes mellitus

## Footnotes

1 Scaling-up Packages of Interventions for Cardiovascular disease prevention in selected sites in Europe and Sub-Saharan Africa: An implementation research – European Commission.

2 Preferred Reporting Items for Systematic Reviews and Meta-Analyses.

3 Appraisal of Guidelines for Research and Evaluation.
