# Supplementary material for: Systematic review of international clinical guidelines for the promotion of physical activity for the primary prevention of cardiovascular diseases
Source: BMC Fam Pract. 2021 May 19;22:97. doi: 10.1186/s12875-021-01409-9 (PMC8136198; doi:10.1186/s12875-021-01409-9)
Supplement: Supplementary file 2 — Additional file 2. [file 12875_2021_1409_MOESM2_ESM.docx]

***SUPPLEMENTARY MATERIAL 2* -** *Recommendation Matrix 1: Core recommendations on content of message to deliver*

| Guideline code, publication year (ref.) | Recommendation | Strength | Description of intervention | Outcome |
| --- | --- | --- | --- | --- |
| CVD 1, 2012 (1) | All adults should be advised to participate in at least 30 minutes of moderate intensity activity on most days or preferably every day of the week. | B  **** | Physical activity = Any bodily movement produced by skeletal muscles that requires energy expenditure. Including occupational and/ or leisure time activity (accumulated bouts of moderate intensity activities, e.g. brisk walking, cycling, taking public transport, household)  Meta-analysis A:  - 22 observational studies, dose-response meta- regression model: non-vigorous PA~mortality  - Interventions~PA duration dose response:  1. 2.5hours/week (30min/day of moderate intensity PA, 5x/week) vs. no activity  2. 7hours/week of moderate intensity PA vs. no activity  Meta-analysis B:  - 38 studies, 3-4 different intensities of regular PA  - Interventions~PA intensity dose response:  1. high vs. moderate/mild intensity,  Men/women  2. mild vs. moderate, men vs. women  Summary of key evidence~ of PA effect on CVD outcomes: **Appendix 3** | Overall outcome of regular PA:  - ↓CVD risk  - ↓CVD risk factors: ↓body fat, ↓BP, ↓LDL-C& triglycerides, ↑HDL-C, ↑insulin sensitivity, protection against T2DM)  PA~CVD risk (inverse relationship):  - ↓Relative risk for CVD 30-40%  - ↓Risk for all-cause mortality 19-33%  Meta-analysis A:  1. ↓mortality risk of 19% (95% CI 15-24)  2. ↓mortality risk of 24% (95% CI 19-29)  🡪 Largest benefit when moving from no activity to low levels of activity  Meta-analysis B:~risk of all-cause mortality  1. - Men: high (RR 0.78; 95% CI 0.72-0.84) vs. mild (RR 0.81; 95% CI 0.75- 0.87)  - Women: high (RR 0.69; 95% CI 0.53-0.90) vs. moderate (RR 0.76; 95% CI 0.66-0.89)  2. Moderate vs. mild: Men RR 0.81; women RR 0.76) |
| CVD 3, 2016 (2) | It is recommended for healthy  adults of all ages to perform at least 150 minutes a week of moderate intensity or 75 minutes a week of vigorous intensity aerobic PA or an equivalent combination thereof, performed in sessions with a duration of at least 10 min/session. Also recommended as part of lifestyle changes for pat with T2DM and all pat with hypertension/high normal BP. | I - A  *** | - Aerobic PA = movements of large muscle mass in a rhythmic manner for a sustained period.  - Everyday activity (active travel, household work, gardening), occupational activity, leisure time activity/exercise (brisk walking, hiking, jogging, cycling, swimming)  - Prescription ~ frequency (at least 3-5x/week, preferably daily)/duration/intensity  - Moderate intensity: at least 30 min/day, 5 days/week  - Vigorous intensity: at least 15 min/day, 5 days/week  - Help pat to set personal goals 🡪 achieve & maintain benefits  - PA activity of choice ~ enjoyed/ included in daily life 🡪 sustainability  - Specifically for DM prevention, combination of both aerobic & resistance exercise is effective | - ↓All-cause & CVD mortality by 20-30% (dose-response)  - Positive effect on CVD risk factors (hypertension, LDL-C, non-HDL-C, body weight, T2DM)  - Lifestyle mgmt (incl. PA) as first measure for prevention of onset /progression of DM & for glycaemia control  - Lifestyle mgmt (incl. regular PA) may be sufficient for pat with high normal & grade 1 hypertension + may reduce dosage of BP-lowering drugs needed for BP control |
|  | For additional benefit in healthy adults, a gradual increase in aerobic PA to 300 minutes a week of moderate intensity, or 150 minutes a week of vigorous intensity aerobic PA, or an equivalent combination thereof is recommended. | I – A  *** | - Longer duration of exercise are proposed for 1. Lipid control (40min/day) & 2. Body weight mgmt (60-90 min/day) |  |
|  | Multiple sessions of PA should be considered, each lasting  ≥10 minutes and evenly spread throughout the week, i.e. on 4–5 days a week and preferably every day of the week | IIa – B  *** |  |  |
| CVD 4, 2017 (3) | Physical activity of at least moderate intensity (e.g. breathing faster than normal) is recommended for the whole population (unless contraindicated by an individual’s condition) | 2. 2++/2+  1+/2++/2+2+  ***** | Rationale:  - PA has both preventive & therapeutic effects on many chronic conditions such as CVD 4£  - PA = Any bodily movement that results in energy expenditure. Categories: occupational (at work), leisure time (non-occupational), exercise (structured & done for a specific reason) & active living (e.g. non-recreational walking, housework, gardening). Four dimensions: duration, frequency, intensity & type. Most acceptable/easy if PA is incorporated into everyday life.  - Reduced cardiorespiratory fitness (ability of the body to use oxygen to do PA, improved by PA) is a risk factor for CVD  - Activities of moderate intensity are protective  - Type of activity is relatively unimportant (occupational vs. leisure time PA)  - No need for continuous PA to have benefit 🡪 longer sessions have no different effect on CHD risk compared with shorter sessions, as long as total energy expenditure is similar  - Potential risk of adverse events associated with vigorous - & high-intensity exercise are extremely low (no significant difference when compared to moderate-intensity PA)  - High levels of total sedentary behavior are associated with higher risk of CVD & mortality  - High levels of sedentary behavior may be associated with additional CVD risk at any level of PA. Undertaking very high levels of PA (>1h/day moderate to vigorous PA) may eliminate the association between excess sitting & CVD risk 🡪 provide general advise to minimize periods of prolonged sitting  In line with national guidance:  - Aim to be active daily. Over a week, activity should add up to at least 150 minutes (2.5 hours) of moderate intensity activity in bouts of 10 minutes or more, or 75 minutes of vigorous-intensity activity.  - Undertake physical activity to improve muscle strength (such as weight training, carrying heavy load, heavy gardening, push-ups or sit-ups) on at least two days a week | 1. PA as independent risk factor:  - Inverse relationship between PA & risk of coronary event  - Active commuting: HR for CVD mortality 1.08 (95% CI 0.95-1.23)  - Running at least 1h/week vs. no running: RR 0.58 (95% CI 0.44-0.77); ARR 0.3%  - High vs. low levels of occupational or leisure time PA: OR 0.51 (95%CI 0.29-0.90)  2. Levels of PA:  - Walking, cycling, gardening 🡪 ↓incidence of myocardial infarction: OR 0.86 (95% CI 0.76-0.97)  - Walking <0.25miles/day vs. >1.5miles/day 🡪 risk of CHD mortality/ morbidity: RR 2.3 (95% CI 1.3-4.1) & ↑incidence of CHD with 2.6% in 2 years  - Dose-response relationship for intensity & duration 🡪 increasing quintiles of energy expenditure (METS): risk of total CVD, ARR 1.00, 0.89, 0.81, 0.78, 0.72 (p<0.001). ↓risk between lowest vs. highest quintiles of PA was 0.5%  3. Vigorous & high-intensity PA vs. moderate –intensity PA:  *Mixed evidence ~outcome*  - ↓BP in hypertension pat  - ↓HbA1c, ↓triglycerides, ↓total cholesterol in T2DM pat  *Potential risks*  - 1h of additional PA in general population: ↑MI AR 2-3/10000 person yrs. & ↑SCD AR 1/10000 person yrs. 🡪 ↓risk in those undertaking habitual PA (by 47% for MI & 30% for SCD; by each additional session per week)  4. Risks of sedentary behavior (highest vs. lowest population groups):  - CVD: RR 2.47 (95% CI 1.44-4.24)  - CVD mortality: RR 1.90 (95% CI 1.36-2.66)  - All-cause mortality: RR 1.49 (95% CI 1.14-2.03)  5. Effects of PA on other key risk factors:  - Aerobic PA 🡪 ↑HDL chol (p<0.05), ↓LDL chol (-5.0%, p<0.05), ↓triglycerides (-3.7%, p< 0.05)  - ↓SBP by 3.8mmHg (95% CI 2.7-5.0) in previously sedentary, normo-/hypertensive adults |
|  | Physical activity may include occupational and/or leisure-time activity and should incorporate accumulated bouts of moderate-intensity activities such as brisk walking |  |  |  |
|  | Those who are moderately active and are able to increase their activity should be encouraged to do so. Activity can be increased through combination of changes to intensity, duration or frequency |  |  |  |
|  | Those who are already moderately active without contraindication can safely be encouraged to undertake vigorous-intensity exercise to achieve additional benefits |  |  |  |
|  | Individuals should be advised to minimize the amount of time spent being sedentary (sitting) over extended periods |  |  |  |
|  | All patients, irrespective of health, fitness or activity level, should be encouraged to increase activity levels gradually | 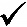  ***** |  |  |
| CVD 5, 2019 (25) | Provide PA advice to every patient with an increased risk of CVD in accordance with the recommendations | N/A |  |  |
|  | Advise at least 150 minutes per week of moderate intensity exercise, such as walking and cycling, spread over several days. Integrate activities into daily life. Longer, more frequent and / or more intensive exercise provides additional health benefits | N/A | Beneficial effects increase as the amount of exercise increases (e.g. build up to 300min/week) | Aerobic exercise:  - ↓ body weight, body fat and waist circumference  - ↓ incidence of stroke, DM |
|  | Advise to avoid a lot of time spent sitting (more than 8 hours a day) | N/A | Excessive sitting is associated with a higher risk of CVD & premature death. |  |
|  | Recommend muscle and bone strengthening activities at least twice a week, for the elderly combined with balance exercises | N/A |  | Aerobic and strengthening exercise:  - ↓ blood pressure  - ↑ insulin sensitivity |
| CVD 6, 2018 (19) | Regular physical activity is recommended to decrease the risk of cardiovascular events and decrease the risk of developing heart failure. | GRADE: A (strong for)  Evidence quality: low  *£* |  | - ↓ Incidence of heart failure |
| CVD 7, 2019 (5) | Adults should engage in at least 150 min per week of accumulated moderate-intensity or 75 min per week of vigorous-intensity aerobic physical activity (or an equivalent combination of moderate and vigorous activity) to reduce CVD risk | COR I – LOE B-NR  *£££* | - Also for afults with T2DM and adults with hypertension (increased physical activity with a structured exercise program)  -Consistent, strong, inverse dose–response relationship between the amount of moderate to vigorous physical activity & incident CVD events and death. Significant benefit observed when comparing those engaging in little or no PA with those performing moderate amounts.  - Shorter durations of exercise seem to be as beneficial as longer ones (eg, ≥10-minute bouts), thus the focus of PA counseling should be on the total accumulated amoun  - Additional reduction in CVD risk is seen in those achieving higher amounts of aerobic PA (>300 min/week of moderate-intensity aerobic PA or 150 min/week of vigorous-intensity aerobic PA) |  |
|  | For adults unable to meet the minimum physical activity recommendations, engaging in some moderate- or vigorous-intensity physical activity, even if less than the recommended amount, can be beneficial to reduce CVD risk | COR IIa – LOE B-NR  *£££* | - No lower limit on the quantity of moderate-to-vigorous PA at which benefits for CVD risk start to accrue.  - Strategies to further increase physical activity in those achieving less than targeted amounts should be implemented |  |
|  | Decreasing sedentary behavior in adults may be reasonable to reduce CVD risk | COR IIb – LOE C-LD  *£££* | - Sedentary behavior = waking behavior with an energy expenditure of ≤1.5 metabolic equivalents while in a sitting or reclining posture  - Increased sedentary behavior is associated with worse health parameters  - Strategies to reduce sedentary behavior, particularly in those not achieving current recommended PA levels, may be beneficial for lowering CVD risk.  - Strength & specificity of the recommendation are limited by uncertainty about the appropriate limits of and optimal approach to modifying sedentary behavior |  |
| LSt 2, 2014 (4) | Advise adults to engage in aerobic physical activity: at least 3 to 4 sessions/week, lasting on average 40 minutes per session, and involving moderate-to-vigorous intensity PA (duration at least 12 weeks) | - NHLBI GRADE: B (mod.)  - NHLBI Evidence statements: CQ3: ES1 (moderate), ES2 (moderate), ES5 (low)  *£*  - ACC/AHA COR: IIa  - ACC/AHA LOE: A  *** | Rationale:  - Association: higher level of PA ~ lower rates of CVD & enhanced longevity  - Inverse dose-response relation: ↑higher levels of PA ≈ ↓rates of CVD (additional health benefits)  - 🡪 Underlying mechanism: PA ~ lipid profile & BP  Evidence Statements (ES) – PA & lipids:  - ES1: Adults, aerobic PA (compared to control)  - ES2: Adults, aerobic PA alone (compared to control)  - ES5: Adults, resistance training(compared to control)  ES – PA & BP:  - ES1: Adults, all BP levels, aerobic PA  - No suff. evidence on resistance for BP reduction | ES – PA & lipids: *Reducing LDL-C and non- HDL-C*  - ES1: ↓LDL-C 3.0 to 6.0 mg/dL  - ES2: ↓non-HDL-C 6mg/dL  - ES5: ↓LDL-C, TG & non-HDL-C by 6 to 9 mg/dL |
|  | Advice adults to engage in resistance training: duration 24 weeks, including ≥3 days/week, 9 exercises, 3 sets & 11 repetitions, intensity 70% of 1-max repetition | - NHLBI GRADE: B (moderate)  - NHLBI Evidence statements: CQ3: ES1 (high)  *£*  - ACC/AHA COR: IIa  - ACC/AHA LOE: A  *** |  | ES – PA & BP: *Lower blood pressure*  - ES1: ↓SBP by 2 to 5 mmHg & ↓DBP by 1 to 4 mmHg |
| OW 2, 2013 (5) | For adults who are overweight or obese, strongly recommend lifestyle change—including reduced energy intake, increased physical activity and measures to support behavioral change. | A  **** | - Lifestyle interventions = multicomponent interventions, most effective when combining nutrition, PA & behavior change  - Delivery in primary HC: GP, practice nurse, other HCP. Brief lifestyle interventions delivered by PN are more cost-effective than delivery by GP  Practice points:  - For adults who are overweight or obese, particularly those who are older than 40 years, there should be an individualized approach to increasing physical activity.  - Individual or group-based psychological interventions may improve the success of weight management programs | - Range of benefits, independent from weight loss: ↑PA without weight loss 🡪 ↓CV risk factors, ↓HbA1c by 0.6 in T2DM pat |
|  | For adults who are overweight or obese, prescribe approximately 300 minutes of moderate intensity activity, or 150 minutes of vigorous activity, or an equivalent combination of moderate intensity and vigorous activities each week combined with reduced dietary intake. | CBR  **** | Studies about association PA & weight loss:  - ↑PA 🡪 range of health benefits even if no weight is lost  - PA has little effect on weight unless it is combined with dietary change  - Dose response amounts of PA~ weight lost  - Maintaining high levels of PA (approximately 60 minutes per day) combines with other behavioral strategies 🡪 ↓weight regain  Cost-resources:  - Brief PA advice delivered in PHC in person/ phone/mail to sedentary people with CVD risk = beneficial + cost-effective  - Exercise referral schemes if no in-house program available = cost-effective option | - PA ~ health benefits: direct /curvilinear  - Greatest benefit in change from doing the least/no PA to doing more  - Frequent PA more effective than only few times/week |
| OW 3, 2014 (6) | Counsel overweight and obese adults with CVD risk factors (high BP, hyperlipidemia, hyperglycemia) that lifestyle changes that produce even modest, sustained weight loss of 3-5% produce clinically meaningful health benefits and greater weight loss produce greater benefits | - NHLBI GRADE: A (strong)  - NHLBI Evidence statements: CQ1: ES1 (high), ES2 (high), ES3 (high)  *£*  - ACC/AHA COR: I  - ACC/AHA LOE: A  *** | Rationale:  - Association: achievement of reduction in body weight with lifestyle interventions ~ CVD risk factors, CVD events, morbidity, mortality  ES1 – weight loss & DM:  Overweight/obese adults, at risk for T2DM, weight loss of 2.5-5.5 kg at ≥2 years, achieved with lifestyle intervention  ES2 – weight loss & lipids:  Overweight/obese adults, with/without elevated CVD risk, dose-response between amount of weight loss achieved by lifestyle intervention & improvement in lipid profile  ES3 – weight loss & BP:  Overweight/obese adults, with elevated CVD risk (incl. T2DM, HT), dose-response between amount of weight loss achieves at up to 3 years by lifestyle intervention & lowering of BP | ES1 – weight loss & DM:  - ↓Risk of developing T2DM by 30-60%  ES2 – weight loss & lipids:  - 3 kg weight loss 🡪 ↓triglycerides of 15 mg/dL  - 5-8kg weight loss 🡪 ↓LDL-C of 5mg/dL & ↑HDL-C of 2-3mg/dL  - <3kg weight loss 🡪 modest/variable improvements in these  ES3 – weight loss & BP:  - At 5% weight loss 🡪 ↓SBP of 3mmHg & ↓DBP of 2mmHg  - At <5% weight loss 🡪 modest/variable reductions in BP |
|  | Advice overweight and obese pat who would benefit from weight loss to participate for ≥6 months in a comprehensive lifestyle program that assists pat in adhering to diet and in increasing PA through the use of behavioral strategies (BT) | - NHLBI GRADE: A (strong)  - NHLBI Evidence statements: CQ4: ES1 (high), ES2 (high)  *£*  - ACC/AHA COR: I  - ACC/AHA LOE: A  *** | Rationale:  - Association: efficacy/effectiveness of comprehensive on-site, high-intensity lifestyle intervention program (components: diet & PA & BT) ~ facilitating weight loss & maintenance of lost weight  ES1 – Description of diet, PA, BT:  All 3 components should be included: 1. Prescription of reduced-calorie diet with energy deficit of ≥500kcal/day. 2. Prescription of increased PA with aerobic PA (e.g. brisk walking) for ≥150min/week (≥30min/day most days of the week). Higher levels of PA (200-300min/week) are recommended to maintain weight loss or minimize weight regain in long term (>1year). 3. Use of structured behavioral therapy including regular self-monitoring of diet/PA/body weight (at least weekly) for therapy adherence + maintaining weight loss  ES2 – Short-/intermediate-/long-term:  - Short-term: Overweight/obese adults, all 3 components, frequent (weekly), on-site treatment by trained interventionistⱡ, group or individual sessions  - Intermediate-term: Providing additional weekly to monthly on-site treatment for another 6 months  - Long-term: After the first year, continuing to provide at least bimonthly intervention contacts | ES2 Short-term:  - Weight loss up to 8 kg in 6 months  - Reductions of 5 to 10% of initial weight  ES2 Intermediate-term:  - Weight loss up to 8kg at 1 year  ES3 Long-term:  - Gradual weight regain of 1 to 2 kg/year from the weight loss achieved at 6 to 12 months.  - Long-term (>1year) weight loss however still remains larger than with usual care |
| OW 4, 2014 (7) | Offer physical activity elements (e.g. home fitness, lifestyle, structured/ supervised PA) that can be combined to produce a caloric deficit leading to weight loss | A  **** | - Pat & provider develop PA plan: activity type, intensity, duration, frequency ~ pat preference, progress, target, abilities  - Home fitness (increasing PA by 30 min/day above baseline, e.g. stairs) 🡪 ↑long-term adherence  - PA combined with caloric restriction for ↑effectiveness  - PA as component of comprehensive lifestyle intervention 🡪 lesse amount of activity is needed for weight loss (because of energy deficit from diet+PA together) | - ↓CVD risk + ↓CVD risk factors  - Weight loss after 15.6 weeks: -2.9kg (PA alone) vs. -11kg (combination PA & diet) |
|  | Offer PA options that include short intermittent bursts (at least 10min) as well as longer continuous exercise | A  **** | - Just as effective as longer duration exercise if total estimated calorie expenditure is the same |  |
|  | Offer, as part of a comprehensive lifestyle intervention, moderate- intensity PA performed for  1. at least 150min/week to result in weight loss  2. at least 200-300min/week to prevent weight regain after initial weight loss | 1. A  2. EO  **** | - Aerobic PA, e.g. brisk walking  - Moderate-intensity = 3.0-5.9 METS  - Dose-response: ↑duration& ↑intensity  🡪 ↑weight loss  - PA = particularly crucial for weight maintenance after initial weight loss | 1. Weight loss & ↓CVD risk Trials: - 6kg in 6 months;  -8kg in 12 months; -16.5 pounds in 12 weeks  2. Longer duration (>200 min/week) or higher intensity PA (>2500kcal/ week) 🡪 improved weight maintenance |
| OW 7, 2015 (8) | Encourage physical activity habits to avoid low energy expenditure. Encourage people to be more physically active and to reduce sedentary behavior. Encourage people to build activity into daily life, developing routines and habits that gradually increase the amount and intensity of activity they do. | NICE  ***** | - Increasing regular walking, particularly brisk walking, or cycling as a form of active travel (to school, work or other local destinations).  - Increasing activities during leisure time and breaks at work or school (including some periods of moderate-to-vigorous physical activity). This could include any form of physical activity, sport or exercise such as walking, cycling, swimming, dancing or gardening.  - Increasing activity as part of daily routines (such as taking regular breaks from sitting at home or work, and taking the stairs instead of the lift).  - Reducing TV viewing and other screen time. Advise people that any strategy that reduces TV viewing and other leisure screen time may be helpful (such as TV‑free days or setting a limit to watch TV for no more than 2 hours a day). | 1. Achieve & maintain energy balance  2. Helps increasing energy expenditure & ↓risk of diseases associated with excess weight (e.g. CVD) |
| LCh 1, 2014 (9) | Advise people to do muscle‑strengthening activities on 2 or more days a week that work all major muscle groups (legs, hips, back, abdomen, chest, shoulders and arms) in line with national guidance for the general population | NICE  ***** | Cost-effectiveness of regular PA for primary prevention of CVD:  - A program to increase PA compared to no program is cost-effective in improving outcomes for people at risk of CVD  - Younger men benefit more from such programs than older men/women  - Unsupervised PA is more cost effective than supervised PA sessions  Moderate intensity activity:  - ↑breathing rate  - ↑heart rate  - ↑warmth, possibly accompanied by sweating  - Can be continued for many minutes without exhaustion feeling  Typical activity pattern:  - Regular active commuting on foot/by bicycle  - Regular work related physical tasks  - Regular household and gardening activities  - Regular active recreation/social sport  Four commonly used methods to ↑PA:  - Brief interventions in PHC  - Exercise referral schemes  - Pedometers/tracking systems  - Community-based exercise programs for walking & cycling | - ↓Total cholesterol  - ↓Triglycerides  - ↓LDL cholesterol  - ↑HDL cholesterol |
|  | Advice about physical activity should take into account the person's needs, preferences and circumstances. Agree goals and provide the person with written information about the benefits of activity and local opportunities to be active. |  |  |  |
| LCh 2, 2014 (10) | All adults should adopt healthy lifestyles to reduce CVD risk, including optimal physical activity | Strong for  *** | - Healthy lifestyle is foundation of primary CVD prevention  - Recommended for all pat, regardless of CVD risk  - Challenges in compliance with positive lifestyle changes 🡪 consider behavior modification & individualizing specific interventions; e.g. patient-friendly tools to assess PA level & track PA  - Well-balanced PA plan: at least 30min, moderate-intensity (e.g. brisk walking), on most (& preferably all) days of the week 🡪 Guidelines on PA for Americans: **Appendix 6** | - Inverse relationship between PA & CVD risk  - ↓LDL-C & ↓non-HDL-C |
| LCh 3, 2018 (54) | Adults should be advised to engage in aerobic physical activity 3-4 sessions per week, lasting on average 40 minutes per session and involving moderate-to vigorous-intensity physical activity. | N/A |  |  |
| LCh 4, 2019 (4) | Physical activity is recommended for 3,5 –7 hours moderately vigorous physical activity per week or 30–60 min most days. | Grade A  *££* | 1. Aerobic physical activity, such as 25–30 km of brisk walking per week (or any equivalent activity) 2. Body weight and physical activity: The intervention should combine diet and exercise; 3. People with dyslipidemia: advice regular physical exercise of moderate intensity for ≥30 min/day, even if they are not overweight | 1. increase HDL-C levels by 0.08–0.15 mmol/L (3.1–6 mg/dL) 2. improvement in physical performance & quality of life, reductions in muscle and bone mass |
| BP 1, 2014 (11) | Offering lifestyle modification interventions for patients with prehypertension or hypertension based on patient indications and preferences as well as assessment of available local resources | Strong for  *** | - Prescription of lifestyle modification by clinician  - For pat with prehypertension (SBP 120-139 mmHg and/or DBP 80-89 mmHg) or hypertension (SBP ≥ 140 mmHg or DBP ≥ 90 mmHg)  - Lifestyle modification strategies: weight reduction (for overweight/obese pat), ↑physical activity, mind-body therapies, dietary pattern (e.g. ↓sodium), ↓alcohol 🡪 combination of lifestyle modifications and/or pharmacologic therapy | - Lower blood pressure  - Other health benefits: ↓diabetes, ↓CVD, ↓blood lipids |
|  | A target for aerobic exercise of 30 to 45 minutes per session, at least 4x/week | Strong for  *** | - Aerobic exercise is fundamental  - Limited evidence for resistance training, but no evidence to exclude it from exercise programs (may confer pat benefits as well) | - Lower blood pressure  - Prevention of large range of health conditions |
| BP 3, 2020 (26) | Weight loss strategies should use a multidisciplinary approach that includes dietary education, increased physical activity, and behavioural intervention | Grade B  *££* |  |  |
|  | For nonhypertensive individuals (to reduce the possibility of becoming hypertensive) or for hypertensive patients (to reduce their BP), prescribe the accumulation of 30-60 minutes of moderate-intensity dynamic exercise (eg, walking, jogging, cycling, or swimming) 4-7 days per week in addition to the routine activities of daily living. Higher intensities of exercise are not more effective | Grade D  *££* |  |  |
|  | For nonhypertensive or hypertensive individuals with SBP/DBP of 140-159/90-99 mm Hg , the use of resistance or weight training exercise (such as free-weight lifting, fixed-weight lifting, or handgrip exercise) does not adversely influence BP | Grade D  *££* |  |  |
| DM 1, 2013 (12) | A structured program of lifestyle modification that includes moderate weight loss and regular physical activity should be implemented to reduce the risk of T2DM in individuals with impaired glucose tolerance (prediabetes, IGT) and impaired fasting glucose (IFG) and A1C 6.0-6.4% | - IGT: Grade A, Level 1A  - IFG: Grade B, Level 2  - A1C 6.0- 6.4%: Grade D, Consensus  *££* | Rationale:  - All DM avoided in white American males through primary prevention 🡪 ↓risk of CVD mortality at population level by 9.0%  - Target population for primary prevention: 1. High-risk individuals (e.g. obesity, IGT); 2. High-risk sub-groups (e.g. low SES); 3. General population  - Variables ~ subsequent T2DM onset: obesity, physical inactivity, IGT & IFG  Trial A & B:  - Comprehensive, sustained program: combination of dietary modification & moderate-intensity PA of at least 150min/week.  - Follow-up of 5.7 more yrs.  Trial C:  - Intensive lifestyle intervention  - Follow-up to 4yrs  Trial D:  - Population: overweight, IFG  - Individual instructions & follow-up support for lifestyle modification from medical staff  - Frequent intervention group (9x/12months) vs. control group (4x/12months)  Trial E:  - Active lifestyle interventions for 6yrs + 14yrs of passive follow-up vs. no treatment | General outcome:  - ↓Individual burden of T2DM  - ↓Individual associated mortality & morbidity  -Public health benefit: ↓CVD rates  Trial A & B:  - Weight loss of 5% of initial body weight  - ↓DM risk of 58% at 4yrs  - Benefits sustained up to 10yrs  Trial C:  - ↓DM risk of 67.4% (p<0.001)  Trial D:  - Incidence of T2DM control: 16.6% (control) --- Incidence of T2DM 12.2% (freq. int.)- HR 0.56 (95% CI 0.36-0.87)  🡪 IGT at baseline:  ↓HR 0.41 (95% CI  0.24-0.69)  🡪 Baseline A1C> 5.6%:  ↓HR 0.24 (95% CI  0.12-0.48)  Trial E:  - Relative risk reduction for incident DM of 43% (95% CI 19-59)  - No identified ↓CVD events/ mortality |
| DM 2, 2014 (13) | To achieve general health benefits: accumulate at least 30 minutes of at least moderate-intensity physical activity on 5 or more days of the week^.^ | NICE  ***** | National recommendations:  - To lose weight: most people may need to do 45–60 min of moderate-intensity activity a day, particularly if they do not reduce their energy intake^.^  - People who have been obese and have lost weight may need to do 60–90 min of activity a day to avoid regaining weight^.^ |  |
| DM 3, 2019 (24) | Moderate-to-vigorous physical activity, notably a combination of aerobic and resistance exercise, for ≥150 min/week is recommended for the prevention and control of DM, unless contraindicated, such as when there are severe comorbidities or a limited life expectancy | I – A  *** | - Lifestyle changes are key to prevent DM and its CV complications.  - Patients with pre-DM and DM should do 2 sessions/week resistance exercise  - Increase activity by any level to yield benefits; e.g. an extra 1000 steps of walking per day as starting point | - PA: delays conversion of IGT to T2DM, and improves glycemic control and CVD complications  - Aerobic and resistance training: improve insulin action, glycaemic control, lipid levels, and BP; reduce HbA1c by ∼0.6% in patients with DM; additive benefit of combined aerobic and resistance exercise. |

ⱡTrained interventionist = mostly HCP who adhere to formal protocols/ lay people that received instruction in weight mgmt protocols (designed by HCP)
