# Supplementary material for: Systematic review of international clinical guidelines for the promotion of physical activity for the primary prevention of cardiovascular diseases
Source: BMC Fam Pract. 2021 May 19;22:97. doi: 10.1186/s12875-021-01409-9 (PMC8136198; doi:10.1186/s12875-021-01409-9)
Supplement: Supplementary file 3 — Additional file 3. [file 12875_2021_1409_MOESM3_ESM.docx]

**SUPPLEMENTARY MATERIAL 3** - Recommendation Matrix 2: Core recommendations on strategies of delivery and implementation of PA message

| Guideline code, publication year (ref.) | Recommendation | Strength | Description of intervention | Outcome |
| --- | --- | --- | --- | --- |
| CVD 1, 2012 (1) | Adults at higher absolute risk of CVD should be given more frequent and sustained lifestyle advice, support and follow-up to achieve behavioral change. | PP  **** | - Lifestyle change interventions to improve CVD outcomes: 1 Cochrane review (55 trials) + 3 trials  - Intervention: Education or counseling with or without pharmacotherapy, >6months duration, to modify >1 CVD risk factor, adults from general population/occupational groups or specific risk factors (diabetes, hypertension, hyperlipidemia, obesity), ≥40yrs old, no evidence of CVD at baseline.  - Some RCT’s used intensive interventions with motivational interviewing based on absolute risk profile, group-based. | BP:  - ↓SBP (WMD -2.71 mmHG; 95% CI -3.49 to  -1.93)  - ↓DBP (WMD -2.13 mmHG; 95% CI -2.67 to  -1.58)  Blood cholesterol: WMD -0.24mmol/L; 95% CI -0.32 to -0.16  Total mortality: OR1.00; 95% CI 0.96-1.05  CHD mortality: OR0.99; 95% CI 0.92 – 1.07  🡪 Ineffective for improving CV outcomes  🡪 However, benefits in total mortality & in combined fatal/nonfatal CV events in people with hypertension (OR 0.78; 95% CI 0.68-0.89) & diabetes (OR 0.71; 95% CI 0.61-0.83)  🡪 Intensive interventions: modest improvements of body weight, waist circumference, BP, overall risk profile |
| CVD 3, 2016 (2) | Established cognitive-behavioral strategies (e.g. motivational interviewing) to facilitate lifestyle change are recommended. | I - A  *** | Lifestyle: Based on long-standing behavioral patterns, maintained by social environment  - Adopting ~ individual + environment + complex/ confusing advice from caregivers  Strategies:  - Individualized counseling ~ pat experiences, thoughts, worries, previous knowledge, everyday life circumstances (= basis for motivation & commitment)  - Decision-making: shared between caregiver & pat/family  - Effective communication facilitates CVD prevention & treatment: friendly / positive interaction / HCP should be trained  - Cognitive behavioral strategies, assessing: pat’s environmental context & thoughts, attitudes, beliefs on perceived ability to change behavior (e.g. previous unsuccessful attempt ~ ↓self-efficacy for future change)  - Combination of realistic goal setting + self-monitoring of chosen behavior (moving forward in small, consecutive steps for changing long-term behavior)  - Principles of effective communication & strategic steps for behavior change: **Appendix 1** | - Cognitive behavioral methods: effective in supporting persons to cope with illness & adopt/adhere to healthy lifestyle changes  - Motivational interviewing: ↑motivation & ↑self- efficacy |
|  | Involvement of multidisciplinary healthcare professionals (e.g. nurses, dieticians, psychologists) is recommended | I – A  *** | - Combining knowledge & skills of caregivers into multimodal behavioral interventions | - To optimize preventive efforts |
|  | Regular assessment and counselling on PA is recommended to promote  the engagement and, if necessary, to support an increase in PA volume over time. | I – B  *** |  |  |
|  | *Media and Education:*  Short term community-based educational programs and wearable devices promoting healthy behaviors, such as walking, should be considered | IIa – C  *** |  | - Recent campaigns from sports medicine societies 🡪 ↑PA prescriptions from GPs |
|  | *Labelling and information:*  Exercise prescription for health promotion by physicians, especially GPs, similar to drug prescription should be considered. | IIa – C  *** |  |  |
| CVD 5, 2019 (3) | Use cognitive behavioral strategies to support lifestyle change, such as motivational interviewing and methods to promote self-management | N/A | Behavioral change starts with exploration of the underlying thoughts about and the motivation for any changes.  Individual goals are determined together with the patient.  Psychosocial factors: Reduction of psychosocial stress, depression and anxiety disorders can stimulate positive behavioral change and improve both quality of life and prognosis |  |
|  | Involve one or more care providers to effect behavioral changes | N/A | Involving a multidisciplinary team helps to achieve the goals. |  |
|  | Use multimodal interventions for people with a very high cardiovascular risk, in which medical treatment is integrated with education about a healthy diet, exercise advice, stress management and discussion about psychological risk factors | N/A | Long-term supervision of behavioral change is more effective than short-term. |  |
| CVD 7, 2019 (4) | Adults should be routinely counseled in healthcare visits to optimize a physically active lifestyle | COR I – LOE B-R  *£££* | - PA assessment & counseling have complementary roles in promoting increased physical activity  - Ascertaining physical activity patterns during a standard clinical visit = first step toward effective counseling & can be accomplished through simple assessment tools (results to be recorded in electronic health record, along with parameters e.g. weight and BP)  - PA counseling by clinicians can result in modest improvements in PA levels (after min 12 for sessions)  - Counseling might include an exercise prescription that consists of recommended frequency, intensity, time (duration), and type of exercise |  |
|  | A team-based care approach us recommended for the control of risk factors associated with CVD | COR I – LOE A  *£££* | - Involvement of multidisciplinary health professionals  - Multifaceted approach, supporting: clinical decision-making (ie, treatment algorithms), collaboration among different clinicians, and patient and family member participation to facilitate the treatment goals of patients  - Use team-based care in: telehealth monitoring, follow-up support aids, patient education  - Meets patient needs better than standard care, especially in low-resource settings and among vulnerable populations | - Greater reduction of CVD risk in patients with hypertension, diabetes, and hyperlipidemia.  - Significant improvements in patient outcomes |
|  | Shared decision-making should guide discussions about the best strategies to reduce CVD risk | COR I – LOE B-R  *£££* | - Occurs when practitioners engage patients in discussions about personalized CVD risk estimates & their implications for the perceived benefits of preventive strategies, including lifestyle habits, goals, and medical therapies.  - Collaborative decisions are more likely to address potential barriers to treatment options, compared with treatment and guidance offered without patient input |  |
|  | Social determinants of health should inform optimal implementation of treatment recommendations for the prevention of CVD | COR I – LOE B-NR  *£££* | - Socioeconomic inequalities: determinants of CVD risk  - Advice should be tailored to: patient’s socioeconomic and educational status, & cultural, work, and home environments  - CVD prevention could benefit from screening low SES. Systems of care should evaluate social determinants of health that affect care delivery for the primary prevention of CVD (eg, transportation barriers, the availability of health services)  - More time may be required to address ASCVD prevention with adults of low health literacy or disadvantaged educational backgrounds. | - Addressing unmet social needs improves mgmt of BP and lipids, which highlights (importance of dietary counseling & encouraging PA)  - Failure to address the impact of social determinants of health impedes efficacy of proven prevention recommendations |
| LSt 1, 2012 (5) | Offer or refer adults who are overweight or obese and have additional CVD risk factors to intensive behavioral counseling interventions to promote a healthful diet and physical activity for CVD prevention | B  **** | Population: adults, primary care settings, overweight/obese + known CVD risk factors (hypertension, dyslipidemia, impaired fasting glucose)  Behavioral counseling interventions:  - Combined counseling on diet & PA  - Intensive: multiple contacts over extended periods (5-16 contacts/9-12 months)  - Format: individual or group, in-person with additional telephone contacts  - Content: focus on behavior change, didactic education + additional support, audit & feedback, problem-solving skills, individualized care plan + at least 2 behavior change strategies (setting specific/proximal goals, providing feedback on progress, providing strategies for self-monitoring, establishing plan for frequency/duration of follow-up, motivational interviewing, building self-efficacy)  - Delivery by specially trained professionals: dietician/nutritionist, physiotherapist/exercise professional, health educator, psychologist  PHC & Community resources:  - Multifaceted approaches with linkage between PHC & community 🡪 ↑effectiveness of interventions  - Effective interactions ~public health/health policy interventions 🡪 support & enhance effectiveness of clinical interventions  - Community-based interventions to promote PA: community-wide campaigns, social support interventions, environmental/policy approach | - Moderate net benefit in target population  - Effective: small but important changes  - Self-reported health behavior: ↑Proportion of people doing moderate- intensity PA 150min/week from 10 to 25%  - ↓CVD events at 6.6 years follow-up (RR 0.62; 95% CI 0.42-0.92)  - Intermediate clinical outcomes after 12-24 months: ↓total cholesterol by 0.14 mmol/L (95% CI 0.07-0.21), ↓LDL by 0.10 mmol/L (95% CI 0.04-0.14), ↓triglycerides by 0.09 mmol/L (95% CI 0.03-0.16); ↓SBP by 2.06 mmHg (95% CI 1.08-3.03), ↓DBP by 1.30 mmHg (95% CI 0.68-1.93); ↓fasting glucose by 0.10 mmol/L (95% CI 0.03-0.18); ↓weight by 0.24 (CI 0.14-0.35); ↓diabetes incidence by RR 0.54 (95% CI 0.34-0.88)  - Different combinations of components are effective  - Strong evidence for programs promoting diet & PA for people at high risk of T2DM 🡪 ↓incidence of new-onset |
| LSt 3, 2014 (6) | Use proven behavior change techniques when designing interventions | NICE  ***** | Providers of behavior change interventions/ programs and intervention designers should:  - Design behavior change interventions to include techniques that have been shown to be effective at changing behavior, incl.: Goals and planning (work with the client to: agree goals for behavior & resulting outcomes, develop action plans, priorities actions, develop coping plans to prevent & manage relapses, consider achievement of outcomes & further goals/plans),  Feedback & monitoring (encourage & support self-monitoring of behavior and its outcomes,  provide feedback on behavior/outcomes), Social support (e.g. advise on/arrange for friends, relatives, colleagues or 'buddies' to provide practical help, emotional support, praise or reward);  - Ensure techniques match service user's needs  - Consider using other evidence-based behavior change techniques that may also be effective  -Define rationale for techniques included  - Ensure novel techniques – or those for which the evidence base is limited – are evaluated  - Consider delivering interventions remotely if there is evidence of efficacy (e.g. telephone, text messaging, apps, internet) |  |
|  | Ensure interventions meet individual needs |  | *Providers of behavior change programs /interventions & trained behavior change practitioners should:*  - Ensure users are given clear information on behavior change interventions/services available & how to use them + help people access  - Ensure services meet users' needs (incl. equity)  - Recognize times when people may be more open to change (e.g. following CVD diagnosis) when it may not be appropriate  *Trained behavior change practitioners should:*  - Assess participants' health in relation to the behavior & type of actions needed (e.g. level/ type of PA recommended is appropriate ~ physical health)  - Ensure intervention intensity matches the person's need for support to change behavior  - Discuss likely impact if behavior is changed (health/wellbeing & the health/ wellbeing of close ones)  - Plan at what point before, during & after behavior change intervention a review will be undertaken to assess progress towards goals + tailor the intervention and follow-up support  - Tailor interventions to meet participants' needs by assessing and then addressing: People's behavior, Participants' physical and psychological capability to make change, context (physical, economic & social environment); How motivated they are to change: if many behaviors need to be changed, assess which one – or ones – the person is most motivated to tackle (see Capability, opportunity and motivation); specific needs |  |
|  | Deliver very brief, brief, extended brief and high intensity behavior change interventions and programs |  | Commissioners /providers of behavior change services should:  - Encourage health, wellbeing & social care staff in direct contact with the general public to use a very brief intervention to motivate people to change behaviors that may damage their health. The interventions should also be used to inform people about services or interventions that can help them improve their general health and wellbeing.  - Encourage staff who regularly come into contact with people whose health/wellbeing could be at risk to provide them with a brief intervention (e.g. low SES)  - Encourage behavior change service providers dealing with the general public to provide an extended brief intervention to people they regularly see for 30 minutes or more who: are involved in risky behaviors, have health problems, are at increased risk of harm, need support to maintain change, have found it difficult to change / have not benefited  - Encourage behavior change service providers & practitioners to provide high intensity interventions (typically these last more than 30 minutes and are delivered over a number of sessions) for people they regularly work with who: have been assessed as being at high risk of causing harm to their health a& wellbeing and/or  have medical condition that needs specialist advice and monitoring (e.g. CVD, T2CM) and/or  have not benefited from lower-intensity interventions |  |
|  | Ensure behavior change is maintained for at least a year |  | Providers & practitioners involved with behavior change programs /interventions should help people maintain behavior change in the long term (more than 1 year) by ensuring they:  - Receive feedback/monitoring at regular intervals for a minimum of 1 year after they complete the intervention (so they can get help if relapse)  - Have well-rehearsed action plans that they can easily put into practice if they relapse  - Have thought about how they can make changes to their own immediate physical environment to prevent a relapse  - Have social support to maintain changes  - Are helped to develop routines that support the new behavior |  |
|  | Commission training for all staff involved in helping to change people's behavior |  | *Commissioners, local education & training boards, managers & supervisors should:*  - Commission training for relevant staff to meet the service specification for any behavior change intervention/program: cover all the various activities (brief & extended); assessment of behaviors & needs; address equity; provide latest available evidence of effectiveness & describe how an intervention works (mechanism of action)  - Ensure training programs on behavior change provide: evidence-based content & training methods; trainers with proven skills, knowledge and experience (competencies); monitoring using competency frameworks or assessment  - Ensure training programs consider: where interventions will be delivered; training participants' characteristics (e.g. SES); whether behavior change is part of participants' main role, integral to their role but not the main focus, or an additional task  - Ensure training includes ongoing professional development (e.g. regular refresher training)  - Ensure training is evaluated in terms of outcomes & process (e.g. participant feedback) |  |
|  | Provide training for behavior change practitioners |  | Providers of behavior change training should:  - Ensure training objectives incl. knowledge/skills (competences) needed for specific interventions  - Ensure practitioners are trained to adopt person-centered approach (with assessment of needs/planning of intervention)  - Ensure behavior change practitioners: understand factors that affect behavior change (incl. psychological, social, cultural & economic);  are aware of behaviors that adversely affect people's health/wellbeing & prevention/ management; can address health inequalities by tailoring interventions to people's specific needs;  are able to assess people's needs & select appropriate evidence-based interventions; know how an intervention works (mechanism of action); recognize specific behavior change techniques; understand how to access/to direct & refer people to specialist support services; understand local policy & demographics  - Ensure behavior change practitioners have the skills to: assess people's behavior (validated assessment tools); communicate effectively (e.g. reflective listening & showing empathy); develop rapport & relationships with service users; develop motivation to change by encouraging/ enabling; deliver relevant behavior change techniques; help prevent & manage relapses  - Ensure behavior change practitioners who provide interventions to groups can: elicit group discussions; provide group tasks that promote interaction/bonding; encourage mutual support within the group  - Give practitioners opportunity to learn how to tailor interventions to meet needs/preferences of different groups  - Ensure trainers have adequate time &resources to assess participants' motivation, skills, confidence & knowledge of specific groups |  |
|  | Provide training for health and social care practitioners |  | All those who train/accredit health & social care professionals should:  - Ensure behavior change knowledge, skills and delivery techniques comprise a formal element of initial training, work placements & ongoing continuous professional development for all those who deliver health and social care services  - Ensure all health and social care professionals can deliver brief intervention (as minimum) |  |
|  | Assess behavior change practitioners and provide feedback |  | *Providers of behavior change training should:*  - Assess ability of trainees to deliver behavior change techniques & tailor to meet needs  *Employers (this includes workplace managers, supervisors and mentors of trainees) should:*  - Ensure behavior change practitioners who have received training are regularly assessed on their ability to deliver behavior change interventions. (very brief to high intensity intervention), reflecting content + including practitioners' ability to provide participants with behavior change techniques & to tailor to participants' needs + service user feedback  *Providers of behavior change training and employers should:*  - Record behavior change sessions as part of the assessment (audio or video recording). If not possible: use reliable observation tool to record intervention (e.g. checklist of key components)  - Obtain consent of the practitioner &service user for all assessments + organization’s confidentiality requirements are met  - Provide practitioners with feedback on their performance (orally & in writing); set jointly agreed goals & action plan + refresher training |  |
| LCh 4, 2019 (7) | A comprehensive patient- and family-centered approach located in one healthcare setting is recommended rather than addressing single risk factors with more than one intervention in different locations. | N/A | Methods for enhancing adherence to lifestyle changes:  1. Explore motivation and identify ambivalence. Weigh pros and cons for change, assess and build self-efficacy and confidence, and avoid circular discussion.  2. Offer support, and establish an alliance with the patient and his/her family.  3.Involve the partner, other household members, or caregiver who may be influential in the lifestyle of the patient.  4.Use the OARS method (Open-ended questions, Affirmation, Reflective listening, Summarizing when discussing behavior changes  5. Tailor advice to an individual patient’s culture, habits, and situation.  6.Use SMART goal setting (negotiate goals for change that are Specific, Measurable, Achievable, Realistic, and Timely). Follow-up on goals and record progress on a shared record. |  |
| OW 1, 2012 (8) | Clinicians should offer or refer patients with a BMI of 30kg/m² or higher to intensive, multicomponent behavioral interventions | B  **** | Most effective interventions:  - Comprehensive  - High intensity (12 – 26 sessions/year) 🡪 ↑treatment intensity 🡪 ↑weight loss  (↓weight loss if <12 sessions)  - Including multiple behavioral mgmt activities, e.g.: group/individual sessions, setting goals, PA sessions, addressing barriers to change, active use of self-monitoring, strategizing how to maintain lifestyle changes | - Weight loss of 6% of baseline weight (4-7kg) in first year (= clinically important) in high intensity intervention, vs. 2.8% of baseline weight (1.5-4kg) if <12 sessions  - In obese pat with elevated plasma glucose levels 🡪 ↓incidence of DM diagnosis by 50% over 2-3 years (NNT 7)  - Improvement in intermediate health outcomes: lipids, BP, waist circumference, glucose tolerance |
| OW 2, 2013 (9) | For adults who are overweight or obese, discuss readiness to change lifestyle behaviors. | D  **** | - HCP need to consider a person’s willingness to undertake the behavioral change required for effective weight management  - More effective when tied to specific behaviors  - Intention, skills/self-confidence, obstacles, positive feelings, self-image/group-norms, encouragement & support  - Training need for HCP (techniques for motivational interviewing) | - Not clear if helpful in predicting weight loss |
|  | For adults, include a self-management approach in weight management programs | C  **** | Supporting behavioral change: Lifestyle education/ information ~ weight loss, lifestyle change, strategies (individualized care planning ~stages of change) + combined with other interventions. Method of delivery: face to face, individually or in group + reinforced by resources (written, web-based, audiovisual materials) 🡪 self-mgmt approach as part of a multicomponent intervention (NOT stand-alone)  - Behavioral change techniques: goal setting, self-monitoring of behavior & progress, stimulus control, cognitive restructuring, problem solving  - Psychological therapies: tailored to the individual  - Other support: reinforce behavioral aspects of care, provide incentives for adherence (rewards for goal achievement), internet-based programs (goal setting, reminders, lifestyle diary), text messages, pedometers  - E.g.: peer-led education on improving self-efficacy in making changes, intensive counselling, short-term goal-setting/ action planning  Practice points:  - Encourage people to make goals for behavioral change  - Regular self-weighing (e.g. weekly) may be a useful component of self-management | - Self-mgmt approach supports lifestyle change + weight loss  - Technology: successful, but not replacement for face to face HC delivery  - Rewards: ↑effectiveness ~ ↑reward size  - ↑frequency of self-weighing 🡪 ↑weight loss + ↓weight gain |
|  | For active weight management in adults, arrange fortnightly review for the first 3 months and plan for continuing monitoring for at least 12 months, with additional intervention as required. | B  **** | Practice points:  - The weight loss plan (type of PA & psychosocial support) should be reviewed after 2 weeks to determine its suitability for that individual and to assess whether it needs to be modified.  - If there is no weight loss (less than 1% body weight or no change in waist circumference) after 3 months of active management, lifestyle behaviors and causes of weight gain should be reviewed (tracking progress towards goals, monitoring changes in risk factors, review plan for care, providing support for motivation). Intensive weight loss interventions may also be considered depending on degree of overweight or obesity and whether comorbidities are present | - Duration over which an intervention is provided & frequency of contact with HCP ~ success of weight loss interventions |
|  | For adults who achieve initial weight loss, strongly recommend the adoption of specific strategies, appropriate to their individual situation, to minimize weight regain. | A  **** | Practice points:  - For long-term weight management, adults can be advised of the importance of taking action (e.g. seeing a health professional) when small amounts of weight (3 kg) have been regained. If there is weight regain, consideration should be given to reassessing energy intake and physical activity, and reintervening with weight loss strategies.  - Long-term weight management may be more successful if it involves a self-management approach, continuing contact with health professionals and behavioral strategies for maintaining motivation.  - Self-management strategies for long-term weight management may include maintaining a healthy lifestyle, identifying ways to manage hunger, setting and reviewing goals, and regular self-weighing. |  |
| OW 3, 2014 (10) | Prescribe on-site, high-intensity (≥14 sessions in 6 months) comprehensive weight loss interventions provided in individual or group sessions by trained interventionistⱡ | - NHLBI GRADE: A (strong)  - NHLBI Evidence statements: CQ4: ES1 (high), ES2 (low), ES3 (moderate to high), ES4 (low)  *£*  - ACC/AHA COR: I  - ACC/AHA LOE: A  *** | ES1 – Primary health care practice setting:  Efficacy/Effectiveness of low-to moderate-intensity comprehensive weight loss programs (lifestyle interventions) in overweight/obese adults within a primary health care practice setting alone  ES2 – On-site vs. electronically delivered:  Effect of characteristics of lifestyle intervention delivery on weight loss & maintenance: comprehensive lifestyle interventions delivered on site by trained interventionistⱡ, initially weekly and then biweekly, group or individual sessions vs. comprehensive interventions delivered by internet or email, including feedback from trained interventionistⱡ  ES3 – Intervention intensity:  - Moderate-intensity: On-site, comprehensive lifestyle interventions, 1 to 2 treatment sessions/month,  - Low–intensity: On-site, comprehensive lifestyle interventions, treatment sessions <1x/month  - High-intensity: On-site, comprehensive lifestyle interventions, ≥14 sessions in 6 months  ES4 – Individual vs. group sessions:  Effect of characteristics of lifestyle intervention delivery on weight loss & maintenance: individual- vs. group-based sessions in high-intensity, comprehensive lifestyle interventions, delivered on-site by trained interventionistⱡ | ES1 – PHC practice:  Not effective compared to usual care  ES2 – On-site/electro.:  Weight loss on-site > weight loss electro.  ES3 - Intensity:  - Moderate: Weight loss of 2 to 4kg in 6 to 12 months (>usual care)  - Low: No weight loss (~ usual care)  - High: Greater net-of-control weight loss (>usual care & low-to moderate intensity interventions)  ES4 –Individual/group:  No ≠ in size of weight loss |
|  | Electronically delivered weight loss programs (including telephone) that include personalized feedback from trained interventionistⱡ can be prescribed for weight loss | - NHLBI GRADE: B (moderate)  - NHLBI Evidence statements: CQ4: ES1 (moderate), ES2 (low)  *£*  - ACC/AHA COR: IIa  - ACC/AHA LOE: A  *** | ES1 – Electronically delivered:  Efficacy/effectiveness of electronically delivered comprehensive interventions in achieving weight loss, developed in academic setting, including frequent self-monitoring of weight/diet/PA, personalized feedback from trained interventionistⱡ  ES2 – Telephone – delivered:  Efficacy/effectiveness of telephone- delivered comprehensive lifestyle interventions, including use of commercially-prepared pre- packaged meals or interactive Web-based program | ES1 – Electronically:  Weight loss up to 5kg at 6 to 12 months (>no/min. intervention offered on Internet/ in print)  ES2 – Telephone:  Weight loss up to 5kg at 6 to 24 months (~ face-to-face counseling, >usual care) |
|  | Commercial-based programs that provide a comprehensive lifestyle intervention can be prescribed for weight loss, provided there is peer-reviewed published evidence of safety/efficacy | - NHLBI GRADE: B (moderate)  - NHLBI Evidence statements: CQ4: ES (low)  *£*  - ACC/AHA COR: IIa  - ACC/AHA LOE: A  *** | ES – Commercial-based:  Efficacy/effectiveness of commercial-based comprehensive lifestyle interventions in achieving weight loss, delivered in-person | ES – Commercial:  - Weight loss of 4.8 to 6.6kg at 6 months with conventional foods (>usual care)  - Weight loss of 6.6 to 10.1kg at 12 months with prepared food (>usual care) |
|  | Advise overweight and obese pat who have lost weight to participate long term (≥1 year) in a comprehensive weight loss maintenance program | - NHLBI GRADE: A (strong)  - NHLBI Evidence statements: CQ4: ES1 (moderate), ES2 (moderate)  *£*  - ACC/AHA COR: I  - ACC/AHA LOE: A  *** | Efficacy/effectiveness of comprehensive lifestyle interventions in maintaining lost weight.  ES1 – Rationale  After initial weight loss, some weight regain can be expected, with greater regain observed over longer periods of time. Continued provision of comprehensive weight loss maintenance program, on-site or by telephone, for periods up to 2.5years after initial weight loss. Optimal duration not yet determined  ES2 – Characteristics  Efficacy/effectiveness of comprehensive lifestyle interventions in maintaining lost weight, overweight/obese adults, high-intensity, long-term | ES1 – Rationale:  Reduces weight gain (>usual care)  ES2 – Characteristics:  35 to 60% maintain a loss of ≥5% of initial body weight at ≥2 years’ follow-up |
|  | For weight loss maintenance, prescribe face-to-face or telephone-delivered weight loss maintenance programs that provide regular contact (at least monthly) with trained interventionistⱡ who helps pat engage in high levels of PA (200-300min/week) monitor body weight regularly (at least weekly) and consume specific diet |  |  |  |
| OW 4, 2014 (11) | Consider providing with information and behavioral counseling regarding healthy diet and physical activity behaviors, in order to maintain or pursue a healthy weight to patients with:  1. normal weight  2. overweight without obesity-associated conditions | 1. C  2. C  **** | Behavioral counseling interventions:  - Activities delivered in primary care setting by clinicians/ related HCP to assist pat in adopting, changing, maintaining health behaviors (e.g. PA)  - Adult pat without CVD, hypertension, hyperlipidemia, diabetes  - Medium- or high intensity. Medium: at least 30min/session, multiple sessions, delivered by health educator/nurse/counselor/psychologist/ exerciser instructor/physiologist instead of GP  - GP can selectively counsel pat or refer them to counseling services within PHC/community. Choice~ CVD risk factors, pat readiness for change, social support, community resources supporting behavioral change, other priorities  - Content: education (health benefits of healthy weight), advice (PA behavior, balanced diet), motivation /encouragement  - Additional for 2.: Establish reasonable weight mgmt goals, realistic expectations & develop plan | 1. Small net benefit on primary prevention of CVD  2. No evidence that weight loss interventions ↓mortality/morbidity from chronic disease among overweight/ non-obese pat |
|  | Offer comprehensive lifestyle interventions (CLI)  1. to achieve weight loss and to improve blood pressure and/or glucose control in overweight pat  2. to overweight pat with dyslipidemia for weight loss and to improve lipid levels  3. to obese pat for weight loss to improve lipid levels, blood pressure and/or glucose control | 1. A  2. B  3. A  **** | Comprehensive lifestyle intervention (CLI) for weight loss (**Appendix 5**):  - Interventions that combine dietary, physical activity & behavioral components  - At least 12 intervention sessions over at least 12 month period | - No evidence that weight loss interventions ↓CVD mortality/morbidity from chronic disease among overweight pat  - Weight loss in overweight/obese population 🡪 ↓obesity-associated conditions e.g. CVD  - Weight loss in overweight/obese population by CLI 🡪 ↓hypertension, ↓T2DM, ↓prediabetes, ↓lipids (strong net benefit) |
|  | Reach a shared understanding with overweight and obese pat about the risks of overweight and obesity and the benefits of weight management | EO  **** | Shared understanding:  - Between clinical team & overweight/obese pat  - Important step in process of helping the pat consider self-mgmt strategies & make informed decisions about treatment  - Methods for reaching SA, based on evidence based principles of health education, health behavior counseling, shared decision-making & motivational interviewing (clinical method for building motivation for behavior change):  1. Ask permission to discuss weight-related health risks & potential benefits/risks of weight loss interventions/mgmt (supports pat autonomy, is pat receptive?)  2. Explore pat understanding, knowledge, beliefs, prior experience, values, influence of family/social network regarding health risks/impact of weight mgmt on health & wellbeing  3. Share information about potential risks based on health status (objective data from medical record)  4. Emphasize the need for ongoing attention/ weight mgmt (lifelong commitment)  5. Provide small amounts of information/advice, tailored to individual values/preferences & easy to understand  6. Use teach-back method to confirm shared understanding  - Strong collaborative pat-provider relationship is built by: effective pat-centered communication, strategies (e.g. open-ended questions, reflective listening, empathy), support of pat autonomy, affirmation of effective coping/self-care strategies  - Pat participation in each element of the plan should be stressed at initiation + periodically over duration of treatment | - Engage dialogue (more than simply informing/ educating pat) 🡪 ↑motivation & ↑working collaboratively with pat to plan next steps  - Strong collaborative pat-provider relationship 🡪 ↑trust + ↑pat attendance & ↑participation in treatment activities & ↑adherence to behavioral elements of intervention |
|  | Perform an in-depth clinical assessment in order to assess the risks and benefits of different weight mgmt treatments and to develop a weight mgmt plan. | EO  **** | Thorough clinical assessment:  - Additional to initial basic assessment  - Essential element of effective behavioral intervention programs for obesity  - For pat willing to commit to weight loss plan  - Assessment of: pat PA & sedentary behavior, social/psychological factors (motivation to change PA & monitoring behaviors) | - Assessment identifies barriers/strengths/ resources that may impact pat participation in weight loss program |
|  | Use motivational interviewing techniques to evoke patient motivation to accept and participate in weight loss treatments | EO  **** | Motivational interviewing (**Appendix 4**):  - For pat not ready to commit to recommended treatment  - Principles & core strategies: 1. Resist directing, 2. Understand the pat’s motivation, 3. Listen with empathy, 4. Empower pat by building confidence, 5. Ask open-ended questions to evoke change talk, 6. Provide affirmation/reflection/summary | - Good evidence: ↑likelihood that pat will follow through with recommendations across wide range of health behaviors |
|  | Offer pat at least 12 contacts within 12 months of a comprehensive lifestyle intervention that combines dietary, physical activity and behavioral strategies | B  **** | Comprehensive lifestyle intervention:  1. Interventions that combine 3 critical lifestyle components (multicomponent): diet, PA & *behavioral component* (setting PA goals, addressing barriers to change, self-monitoring, strategize for maintaining lifestyle changes, stimulus control, positive reinforcement, stress mgmt, problem solving, cognitive restructuring)  2. Intensive: At least 12 intervention sessions over 12 month period  3. Delivery methods in primary care setting: in-person, telephone, web-based interface by trained counsellors/coaches (physician and/or non-physician)/referral to evidence-based commercial programs (with primary care clinician follow-up & support) | 1. No single type of behavioral strategy is superior to others 🡪 multimodal strategies for better outcome  2. - Intensive interventions 🡪 significantly greater weight loss than less intensive interventions  - Total weight loss intensive group of 4-7kg vs. 1.5-4kg in less intensive group  3. - Effective impact of comprehensive lifestyle interventions in primary care settings  - Delivery by non-physician staff or referral commercial program 🡪 clinically significant weight loss more likely than delivery by physicians alone |
|  | Assess adherence to the weight loss program 1-2x/month by measuring the pat’s weight and providing feedback and ongoing support. | EO  **** | Frequent follow-up (intensity):  - Provides pat to address challenges of adopting PA & other behavioral strategies  - Frequent team contacts provide opportunities to review pat’s progress & assess pat’s experience in making desired changes in PA & other self-mgmt behaviors  - If motivation is waning to meet behavioral goals 🡪 give specific attention to these elements  - If pat continues to struggle to meet short-term goals, consider increasing the intensity |  |
|  | Offer pat who have met their weight loss goals a comprehensive maintenance program consisting of all behavioral components and ongoing support | B  **** | - Focus of weight mgmt is preventing weight regain by maintenance of PA & other self-mgmt behaviors that contributed to initial weight loss  - Extended follow-up contact + measuring of weight at each routine visit or at least 2x/year | - Follow-up contact 🡪 82.7% maintenance of initial weight loss vs. 33.3% if no follow up at 12 months post-treatment |
|  | Offer comprehensive lifestyle interventions for weight loss  1. in either individual or group setting  2. telephone based, either as an alternative or an adjunct to face-to-face intervention  3. internet-based, either as an alternative or an adjunct to face-to-face intervention | 1. B  2. B  3. I  **** | Format of delivering:  1. Often delivered in group programs  2. Telephone as an alternative for those who cannot participate in F2F interventions or as a supplement to F2F sessions  3. Internet or other electronic health delivery systems offer alternatives + ↑opportunity to asynchronous intervention/↑accessibility to intervention & trained interventionist | 1. Treatments utilizing either group or individual F2F formats are effective  2. Telephone-based interventions are effective for achieving weight loss |
| OW 5, 2014 (12) | Managers and health professionals in all primary care settings should ensure that preventing and managing obesity is a priority at both strategic and delivery levels. Dedicated resources should be allocated for action. | NICE  ***** | Strategy:  - In their role as employers (senior mgers, budget holders) should set an example in developing public health policies to prevent & manage obesity by following existing guidance and & local obesity strategy, e.g. policies, facilities and information that promote PA,  - All primary care settings (PCS) should ensure that systems are in place to implement local obesity strategy (e.g. training of HCP, multidisciplinary teams)  - All PCS should: address training needs of staff involved, allocate adequate time & space, develop multidisciplinary teams  - Local health agencies should identify appropriate HCP & provide training in: health benefits & potential effectiveness of interventions to prevent obesity/ increase PA, best practice approaches in delivering such interventions + tailoring support to meet long term needs, use motivational/counselling techniques, barriers to health professionals providing support/advice, concerns about effectiveness of interventions, pat’s receptiveness & ability to change, impact of advice pat-HCP relationship  Delivery:  - Interventions to ↑PA: activities fitting into everyday life (e.g. walking), tailored individual preferences/circumstances & improve belief in their ability to change (e.g. by verbal persuasion, modelling exercise behavior, discussing positive effects). Ongoing support (incl. written materials): in person, by phone, mail, internet  - Interventions (incl. promotional, awareness-raising activities) as part of a long-term, multicomponent intervention & targeted follow-up with different population groups  - HCP should discuss weight, & PA times when weight gain is more likely e.g. menopause/ smoking cessation  - Community programs to prevent obesity/↑PA ~concerns of local people from the outset (e.g. availability of services, cost of changing behavior, expectation, dangers, confusion)  - HCP should support & promote community schemes/facilities that improve access to PA, combined with tailored information based on local needs  - HCP should support & promote behavioral change programs along with tailored advice to help people who are motivated to change  - Occupational HCP & public health practitioners in partnership with local businesses to support implementation of workplace programs |  |
| OW 6, 2015 (13) | For adults who are obese (BMI 30-39.9) and at high risk of T2DM, practitioners should offer or refer to structured behavioral interventions aimed at weight loss. | Strong, moderate-quality evidence  *** | Structured interventions = Intensive behavioral modification programs involving several sessions over weeks to months, including behaviorally based interventions focused on diet, exercise or lifestyle changes, alone or in combination. Lifestyle changes include counseling, education or support, and/or environmental changes in addition to changes in exercise and/or diet.  Evidence background:  - High risk of T2DMn defined by impaired glucose tolerance (6 studies) or by validated risk assessment tools (1 study) | Weight-related outcomes by intervention participants at high risk of T2DM vs. controls:  - Less likely to be diagnosed with new-onset T2DM (incidence 11 vs. 20%; RR 0.6; 95% CI 0.4 to 0.7; absolute risk reduction 8.9%)  - Risk of T2DM 34% lower with intensive lifestyle changes on long-term (10yr) (95% CI 24 to 42) |
|  | For adults who are overweight or obese, practitioners should offer or refer to structured behavioral interventions aimed at weight loss. | Weak, moderate-quality evidence  *** | Structured interventions: as defined in previous recommendation  Heterogeneous intervention characteristics:  - Provider discipline, length, format  - Best outcome with: >12 months duration, including diet and/or exercise and/or lifestyle components, group and individual sessions  Evidence background:  - Based on meta-analysis of trials with behavioral interventions (diet/exercise/ lifestyle components  - Population: Participants with/without diabetes/other chronic conditions (applies to all populations other than those at high risk of T2DM)  - Participation in diet alone/diet and exercise/lifestyle interventions led to greater reduction in weight: 4.7kg (95% CI -6.2 to -3.2), 3.8kg (95% CI -5.5 to -2.2), 2.5kg (95% CI -3.5 to -1.5kg)  - Exercise alone was not associated with weight loss. Also no difference based on duration of PA. | Intervention participants vs. controls: 1. Weight:  - ↓Weight by 3.1kg more (95% CI -3.9 to -2.4)  - ↓BMI by an additional 1.09 (95% CI -1.4 to -0.8)  - ↓Waist circumference by an additional 3.05 cm (95% CI -3.9 to -2.2)  - ↓Body fat by 1.3% more  - More likely to lose ≥5% (NNT 9; RR 1.8; 95% CI 1.4-2.3)  - More likely to lose ≥10% of total body weight (NNT 12; RR 2; 95% CI 1.3-3.2)  2. Lipids:  - ↓Total cholesterol by 0.1mmol/L (95% CI -0.2 to -0.03)  - ↓LDL by 0.14mmol/L (95% CI -0.3 to -0.0)  3. Diabetes:  - ↓fasting glucose by 0.14mmol/L (95% CI -0.2 to -0.05)  4. BP:  - ↓SBP by 1.8mmHg (95% CI -2.6 to -0.9)  - ↓DBP by 1.6 mmHg (95% CI -2.3 to -- 0.9) |
| OW 7, 2015 (14) | Encourage self-monitoring. Encourage habits that may help people to monitor their weight or associated behaviors. | NICE  ***** | - Checking their physical activity level (for example, by noting down activities, or using a pedometer or an app to track physical activity) |  |
|  | Clearly communicate the benefits of maintaining a healthy weight. Clearly communicate the broad range of benefits of maintaining a healthy weight through being more physically active and improving dietary habits (including 'non health' benefits as well as improvements to health). |  | - ↑enjoyment from shared, social physical activities  - ↓risk of developing diseases associated with excess weight ( coronary heart disease, hypertension, stroke, T2DM)  - ↑mental wellbeing  - ↓breathless, ↑fitness and other benefits from increased physical activity that are independent of weight |  |
|  | Clearly communicate the benefits of gradual improvements to physical activity and that even small, gradual improvements to physical activity are likely to be helpful. |  | - Improving dietary habits and being physically active are as important for people who are currently a healthy weight as for people who are already overweight  - No single physical activity, food or drink will maintain a healthy weight – a combination of actions is needed |  |
|  | Tailor messages for specific groups |  | Tailor messages (e.g., for different socioeconomic groups). Ensure all messages are clear, consistent, specific and non-judgmental. |  |
| LCh 1, 2014 (15) | Communication about risk assessment and treatment in order to encourage the person to participate in reducing their CVD risk | NICE  *** | - Find out what, if anything, the person has already been told about their CVD risk and how they feel about it  - Explore the person's beliefs about what determines future health (this may affect their attitude to changing risk)  - Assess their readiness to make changes to their lifestyle (diet, physical activity, smoking and alcohol consumption), to undergo investigations and to take long‑term medication  - Assess their confidence in making changes to their lifestyle, undergoing investigations and taking medication  - Inform them of potential future management based on current evidence and best practice  - Involve them in developing a shared management plan  - Check with them that they have understood what has been discussed |  |
|  | Recognize that people may need support to change their lifestyle. To help them do this, refer them to programs such as exercise referral schemes. |  |  |  |
| BP 1, 2014 (16) | Offering a multi-modal approach to adherence interventions, which could include tele monitoring, multi-disciplinary group medical appointments (e.g. shared medical appointments), case management (by pharmacists, nurses, social workers), patient and provider education, behavioral therapy | Weak for  *** | - Combination of nurse administered behavioral mgmt & medication mgmt interventions  - Multidisciplinary approach (e.g. GP, pharmacist, nurse)  - Nurse- or pharmacist directed case mgmt, using structured follow-up & monitoring (e.g. tele monitoring, electronic web-based monitoring/communication, in-person/telephone-based follow-up) | - Sustained improvement in blood pressure (decrease)  - Adherence to therapy  - Adequate control of hypertension  - Most effective in “high risk” pat (high baseline BP + poor baseline control rates) |
|  | The use of a self-monitoring device (e.g. pedometer, mobile apps) to increase adherence to physical activity | Weak for  *** | - To maximize benefits from physical activity; compared to pat education/prescription for exercise alone  - Structured & supervised exercise programs; compared to counseling/usual care | - ↑Adherence to exercise regimens  - ↑Reduction in BP |
|  | Pat be seen within one month of initiation of lifestyle or pharmacological therapy to determine adequacy of hypertension control, degree of pat adherence, presence of adverse effects | Weak for  *** | - Objectives: stressing the importance of diagnose to pat, helping HCP to assess pat adherence/side effects/challenges  - Initiation of treatment & follow-up of resistant cases/ therapy titration by credentialed provider (clinician). Follow up by other HCP than clinician for pat education, assessment of adherence/potential adverse effects | - Assess full antihypertensive effect |
|  | Once pat’s BP is controlled, at least annually follow-up is suggested (more frequently as indicated), depending on pat preference | Weak for  *** | - No definitive evidence on what duration between follow-up visits  - At least annual follow-up visit for well-controlled, uncomplicated pat  - Instruct pat to contact HCP earlier in case of: side effects/other challenges with continuing therapy | - Maintain optimal blood pressure |
| BP 2, 2014 (17) | Team-based care is recommended to improve blood pressure control | Strong for  *** | Team-based care:  - Health systems/organizational intervention  - Multidisciplinary team: adding new staff or changing roles of existing staff to work with PHCP  - Team = pat + primary care provider + other professionals (nurse, pharmacist, dietitian, social worker, community health worker) 🡪 working in collaboration  - Process support & sharing of responsibilities 🡪 complementing activities of PHCP  - Including: communication/coordination among various team members, use of evidence-based guidelines, regular/structured proactive follow-up mechanisms, engaging pat in own care by education & adherence/self-mgmt support (incl. tools/resources for health behavior change: developing pat knowledge/skills & improving attitudes/health behavior)  - Provide community-based support through community health workers 🡪 assisting HCP & pat by serving as liaisons tot the HC system & lay educators | Strong/sufficient evidence for:  - Cost effective  - Effectiveness in ↑proportion of pat with controlled BP, ↓SBP, ↓DBP + improvements in diabetes & lipid outcomes |
| DM 2, 2014 (18) | Interventions for communities at high risk of type 2 diabetes | NICE  ***** | Who should take action:  Local PHS in partnership with other local authority departments, voluntary/non-profit sector, primary health care providers  What action to take:  - Work in partnership to develop cost-effective PA interventions (~ culture, low SES)  - Identify success criteria for evaluation of interventions  - Identify skills gaps & train/recruit staff  - Identify & address barriers to participation (communication strategies ~ target audience's language & information requirements)  - Use community resources to ↑awareness & ↑accessibility (e.g. community organizations, leaders, media, events, festivals specifically aimed at low SES groups, involve existing community & social groups)  - Use community links, outreach projects and lay or peer workers (from lower SES groups) to deliver interventions.  - Train lay & peer workers in how to plan, design and deliver community-based health promotion activities. Training: based on proven training models & evaluation techniques, give participants the chance to practice new skills in the community, encourage them to pass on their knowledge to peers.  - Lay/peer workers & HCP should identify and encourage 'community champions' (e.g. religious and community leaders) to promote PA  - Encourage lay & peer workers to get other members of their community involved  - Ensure lay & peer workers are part of a wider team led by HCP. Involve them in planning, design and delivery of credible appropriate messages (including helping people to develop practical skills to adopt healthy lifestyle). Mgmt & supervision should be provided by HCP leading these teams.  - Assessment of a high-risk community should be culturally appropriate  - Linked to effective services and interventions |  |
|  |  |  |  |  |
|  | Conveying messages to the local population |  | Who should take action:  Local PHS in partnership with other local authority departments, voluntary/non-profit sector, primary health care providers  What action to take:  - Work with local practitioners, role models & peers to tailor national messages for the local community about preventing CVD & T2DM  - Healthier lifestyle messages: consistent, clear, culturally appropriate, integrated within other local health promotion campaigns/interventions. - Address misconceptions in the local community that could act as a barrier to change.  - Ensure messages & information are disseminated locally to groups at higher risk of type 2 diabetes than the general population (low SES). Use local newspapers, online social media, local radio channels targeted at these groups. Use local shops and businesses, community workers and groups, social establishments, educational institutions, workplaces, places of worship and local health care establishments  - Offer communities support to improve their PA levels, and ensure awareness of importance |  |
|  | Promoting physical activity: local action |  | Who should take action:  Local PHS in partnership with other local authority departments, voluntary/non-profit sector, primary health care providers, schools, fitness industry, employers, public transport, …  What action to take:  - Use existing mechanisms for local planning to: prioritize the need for people to be physically active as a routine part of their daily life (e.g. local infrastructure), provide open or green spaces to give people local opportunities for walking & cycling, make sure local facilities/ services are easily and safely accessible by transport involving physical activity, provide for PA in safe/accessible locations, encourage people to be physically active inside buildings (stairs)  - Achieve the national recommended levels of PA by including activities such as walking, cycling or climbing stairs as part of everyday life.  - Assess PA opportunities needed locally (~social norms, family practices)  - Map PA opportunities against local needs and address any gaps.  - Commissioned leisure services affordable & acceptable (~ SES, culture)  - Information on local, affordable, practical and culturally acceptable opportunities for PA  - Local employers to develop policies to encourage PA in employees  - Basic training for professional fitness instructors covers: the role of PA in improving health, how to get marginalized groups involved & cultural issues that may prevent them from participating |  |
|  | Training those involved in promoting healthy lifestyles |  | Who should take action:  National & local PHS in partnership with other authority departments, voluntary/non-profit sector, commercial sector, education/leisure services, royal/professional associations  What action to take:  - Training programs for those responsible for & involved in, promoting healthy lifestyle, incl.: diversity (culture, SES), non-judgmental attitude, meeting needs (language & literacy), how to identify communities at increased risk, strategies for changing behavior, how to provide advice on PA in relation to CVD  - Give time/support to develop & maintain skills  - Monitor HCP’s knowledge & awareness of how to encourage people to adopt a healthy lifestyle (personal development plans, annual reviews), keep knowledge and practical skills up-to-date.  - Training programs for all HCP: incorporate knowledge & skills to ensure health promotion interventions are culturally sensitive, cover PA in relation to prevention of CVD & risk factors, focused/structured & based on proven models and evaluation techniques, opportunities to practice new skills in the community, sharing of knowledge among colleagues, up-to-date information on topics such as PA |  |

ⱡTrained interventionist = mostly HCP who adhere to formal protocols/ lay people that received instruction in weight mgmt protocols (designed by HCP)

# References

1. National Vascular Disease Prevention Alliance. Guidelines for the management of absolute CVD risk [Guideline]. 2012 [Available from: <https://www.strokefoundation.com.au/~/media/strokewebsite/resources/treatment/absolutecvd_gl_webready.ashx?la=en>.

2. Piepoli MF, Hoes AW, Agewall S, Albus C, Brotons C, Catapano AL, et al. 2016 European Guidelines on cardiovascular disease prevention in clinical practiceThe Sixth Joint Task Force of the European Society of Cardiology and Other Societies on Cardiovascular Disease Prevention in Clinical Practice (constituted by representatives of 10 societies and by invited experts)Developed with the special contribution of the European Association for Cardiovascular Prevention &amp; Rehabilitation (EACPR). European heart journal. 2016;37(29):2315-81.

3. Practitioners N-DCoG. Cardiovascular risk management (M84) Netherlands: NHG; 2019 [updated June 2019. Version 4.0:[Available from: <https://richtlijnen.nhg.org/standaarden/cardiovasculair-risicomanagement#volledige-tekst-literatuur>.

4. Arnett DK, Blumenthal RS, Albert MA, Buroker AB, Goldberger ZD, Hahn EJ, et al. 2019 ACC/AHA Guideline on the Primary Prevention of Cardiovascular Disease: A Report of the American College of Cardiology/American Heart Association Task Force on Clinical Practice Guidelines. Circulation. 2019;140(11):e596-e646.

5. National Guideline C. Behavioral counseling interventions to promote a healthful diet and physical activity for cardiovascular disease prevention in adults: U.S. Preventive Services Task Force recommendation statement. 2012.

6. NICE - National Institute for Health + Care Excellence. Behaviour change: individual approaches (PH49) [Guideline]. 2014 [Available from: <http://guidance.nice.org.uk/PH49>.

7. Mach F, Baigent C, Catapano AL, Koskinas KC, Casula M, Badimon L, et al. 2019 ESC/EAS Guidelines for the management of dyslipidaemias: lipid modification to reduce cardiovascular risk: The Task Force for the management of dyslipidaemias of the European Society of Cardiology (ESC) and European Atherosclerosis Society (EAS). European heart journal. 2019;41(1):111-88.

8. National Guideline C. Screening for and management of obesity in adults: U.S. Preventive Services Task Force recommendation statement. 2012.

9. National Guideline C. Clinical practice guidelines for the management of overweight and obesity in adults, adolescents and children in Australia. 2013.

10. National Guideline C. 2013 AHA/ACC/TOS guideline for the management of overweight and obesity in adults: a report of the American College of Cardiology/American Heart Association Task Force on Practice Guidelines and The Obesity Society. 2014.

11. National Guideline C. VA/DoD clinical practice guideline for screening and management of overweight and obesity. 2014.

12. NICE - National Institute for Health + Care Excellence. Obesity (CG43) [Guideline]. 2014 [Available from: <http://guidance.nice.org.uk/CG43>.

13. National Guideline C. Recommendations for prevention of weight gain and use of behavioural and pharmacological interventions to manage overweight and obesity in adults in primary care. 2015.

14. National Guideline C. Maintaining a healthy weight and preventing excess weight gain among adults and children. 2015.

15. NICE - National Institute for Health + Care Excellence. Cardiovascular disease: risk assessment and reduction, including lipid modifiation (cg181) [Guideline]. 2014 [Available from: <http://guidance.nice.org.uk/CG67>.

16. National Guideline C. VA/DoD clinical practice guideline for the diagnosis and management of hypertension in the primary care setting. 2014.

17. National Guideline C. Team-based care to improve blood pressure control: recommendation of the Community Preventive Services Task Force. 2014.

18. NICE - National Institute for Health + Care Excellence. Preventing type 2 diabetes - population and community interventions (PH35) [Guideline]. 2011 [Available from: <http://guidance.nice.org.uk/PH35>.
