# Supplementary material for: Systematic review of international clinical guidelines for the promotion of physical activity for the primary prevention of cardiovascular diseases
Source: BMC Fam Pract. 2021 May 19;22:97. doi: 10.1186/s12875-021-01409-9 (PMC8136198; doi:10.1186/s12875-021-01409-9)

**SUPPLEMENTARY MATERIAL 4 - GRADING: Background information about grading of levels of evidence & grades of recommendations, reported from the included guidelines in Recommendations matrices 1 and 2**

*** Definitions**

Guyatt, G. H., Oxman, A. D., Vist, G. E., Kunz, R., Falck-Ytter, Y., Alonso-Coello, P., Schünemann, H. J. & the GRADE Working Group. (2008). GRADE; An emerging consensus on rating quality of evidence and strength of recommendations. *BMJ, 336*, 924-926.


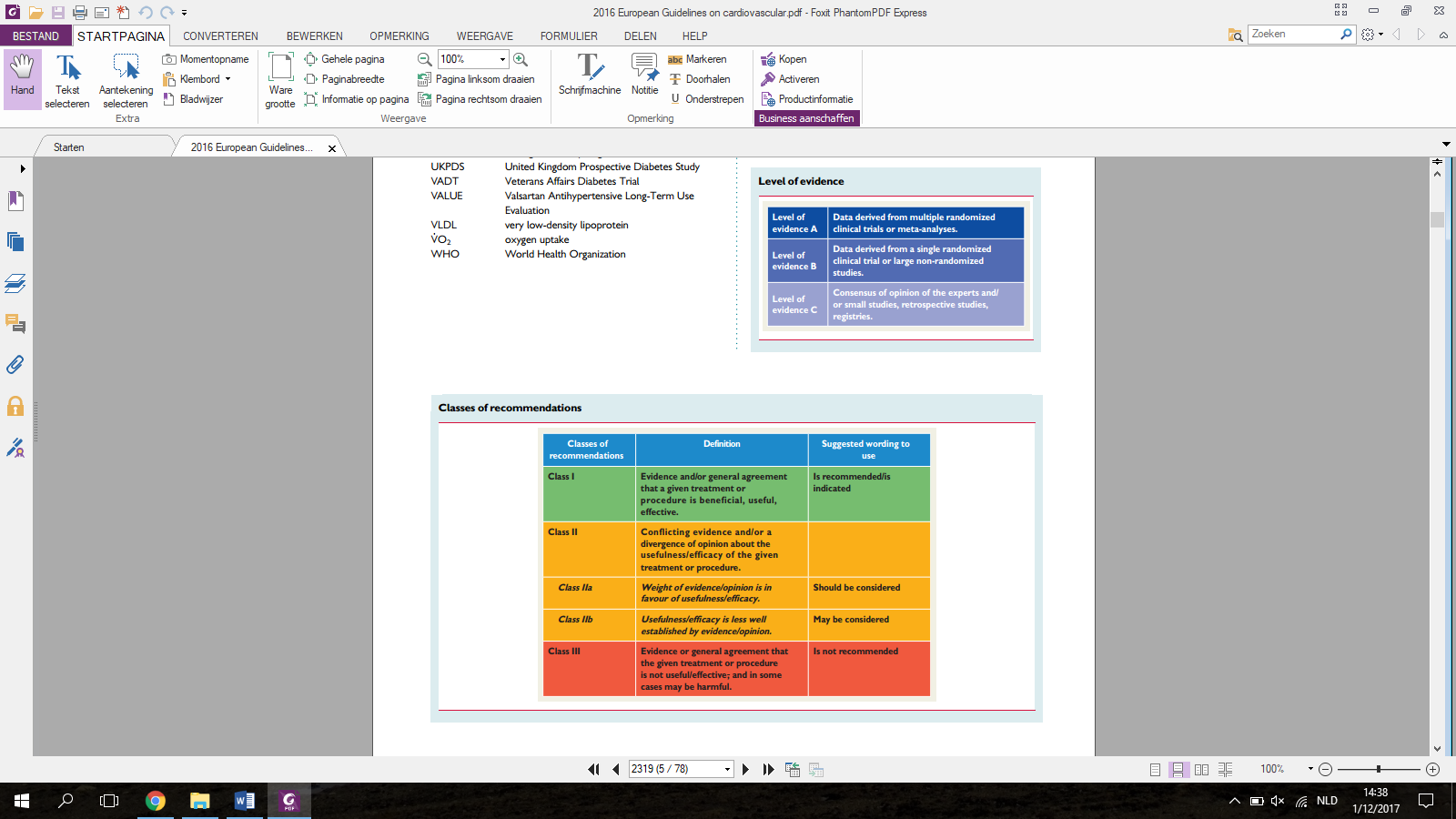


**
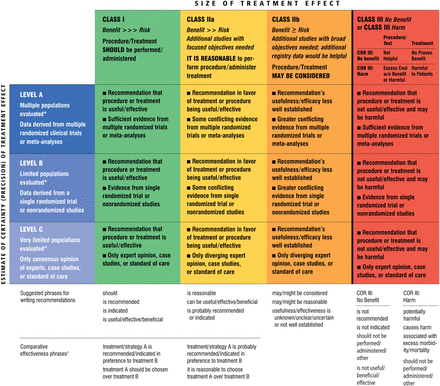
**

**** Definitions**


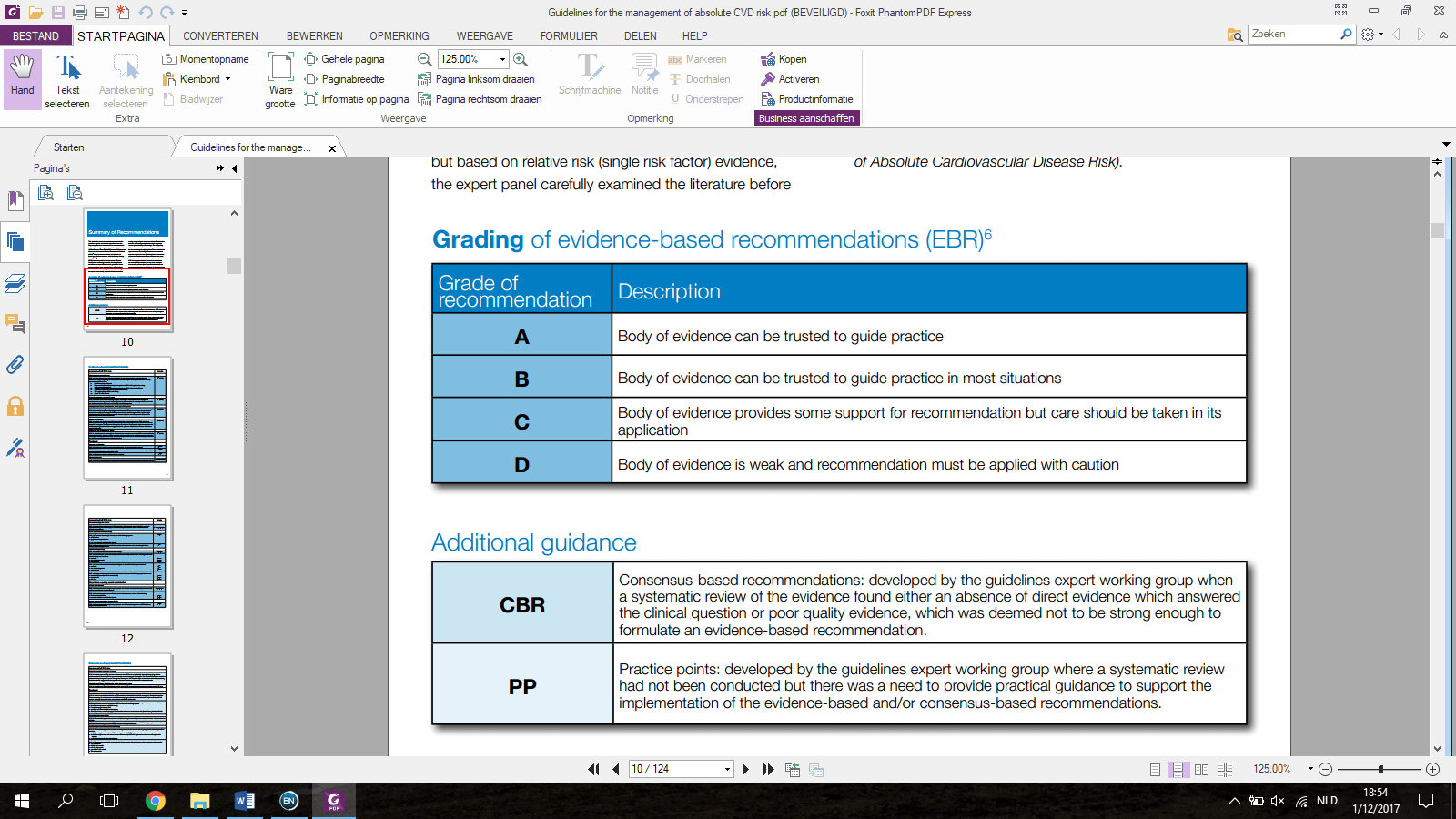


***** Definitions**


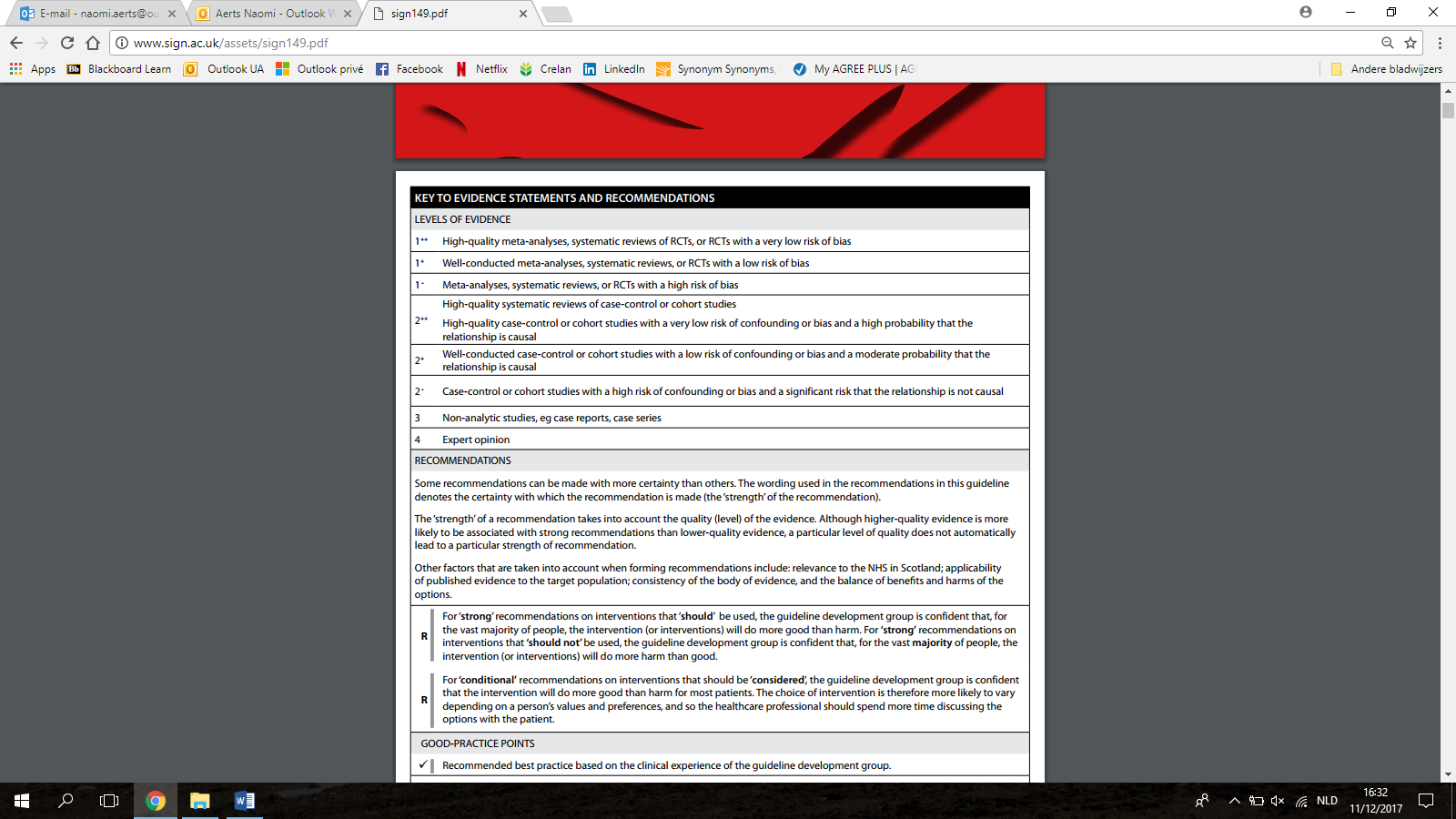


In **NICE guidelines** using this grading system, only 1++, 1+, 2++, 2+ level recommendations were reported.

**£ Definitions**

**NHLBI Grading of the Strength of Recommendations**

| **Grade** | **Strength of Recommendation^∗^** |
| --- | --- |
| A | **Strong recommendation** There is high certainty based on evidence that the net benefit**†** is substantial. |
| B | **Moderate recommendation** There is moderate certainty based on evidence that the net benefit is moderate to substantial, or there is high certainty that the net benefit is moderate. |
| C | **Weak recommendation** There is at least moderate certainty based on evidence that there is a small net benefit. |
| D | **Recommendation against** There is at least moderate certainty based on evidence that there is no net benefit or that risks/harms outweigh benefits. |
| E | **Expert opinion (“There is insufficient evidence or evidence is unclear or conflicting, but this is what the Work Group recommends.”)** Net benefit is unclear. Balance of benefits and harms cannot be determined because of no evidence, insufficient evidence, unclear evidence, or conflicting evidence, but the Work Group thought it was important to provide clinical guidance and make a recommendation. Further research is recommended in this area. |
| N or I | **No recommendation for or against (“There is insufficient evidence or evidence is unclear or conflicting.”)** Net benefit is unclear. Balance of benefits and harms cannot be determined because of no evidence, insufficient evidence, unclear evidence, or conflicting evidence, and the Work Group thought no recommendation should be made. Further research is recommended in this area. |

- In most cases, the strength of the recommendation should be closely aligned with the quality of the evidence; however, under some circumstances, there may be valid reasons for making recommendations that are not closely aligned with the quality of the evidence. Those situations should be limited and the rationale explained clearly by the Work Group.

| **Type of Evidence** | **Quality Rating^∗^** |
| --- | --- |
| Well-designed, well-executed**†** RCT that adequately represent populations to which the results are applied and directly assess effects on health outcomes. Meta-analyses of such studies. Highly certain about the estimate of effect. Further research is unlikely to change our confidence in the estimate of effect. | High |
| RCT with minor limitations**‡** affecting confidence in, or applicability of, the results. Well-designed, well-executed nonrandomized controlled studies**§** and well-designed, well-executed observational studies**‖**. Meta-analyses of such studies. Moderately certain about the estimate of effect. Further research may have an impact on our confidence in the estimate of effect and may change the estimate. | Moderate |
| RCT with major limitations. Nonrandomized controlled studies and observational studies with major limitations affecting confidence in, or applicability of, the results. Uncontrolled clinical observations without an appropriate comparison group (e.g., case series, case reports). Physiological studies in humans. Meta-analyses of such studies. Low certainty about the estimate of effect. Further research is likely to have an impact on our confidence in the estimate of effect and is likely to change the estimate. | Low |

- In some cases, other evidence, such as large all-or-none case series, can represent high- or moderate-quality evidence. In such cases, the rationale for the evidence rating exception should be explained by the Work Group and clearly justified.
- † “Well-designed, well-executed” refers to studies that directly address the question; use adequate randomization, blinding, and allocation concealment; are adequately powered; use intention-to-treat analyses; and have high follow-up rates.
- ‡ Limitations include concerns with the design and execution of a study that result in decreased confidence in the true estimate of the effect. Examples of such limitations include but are not limited to: inadequate randomization, lack of blinding of study participants or outcome assessors, inadequate power, outcomes of interest that are not prespecified for the primary outcomes, low follow-up rates, and findings based on subgroup analyses. Whether the limitations are considered minor or major is based on the number and severity of flaws in design or execution. Rules for determining whether the limitations are considered minor or major and how they will affect rating of the individual studies will be developed collaboratively with the methodology team.
- § Nonrandomized controlled studies refer to intervention studies where assignment to intervention and comparison groups is not random (e.g., quasi-experimental study design).
- ‖ Observational studies include prospective and retrospective cohort, case-control, and cross-sectional studies.

**££ Definitions**


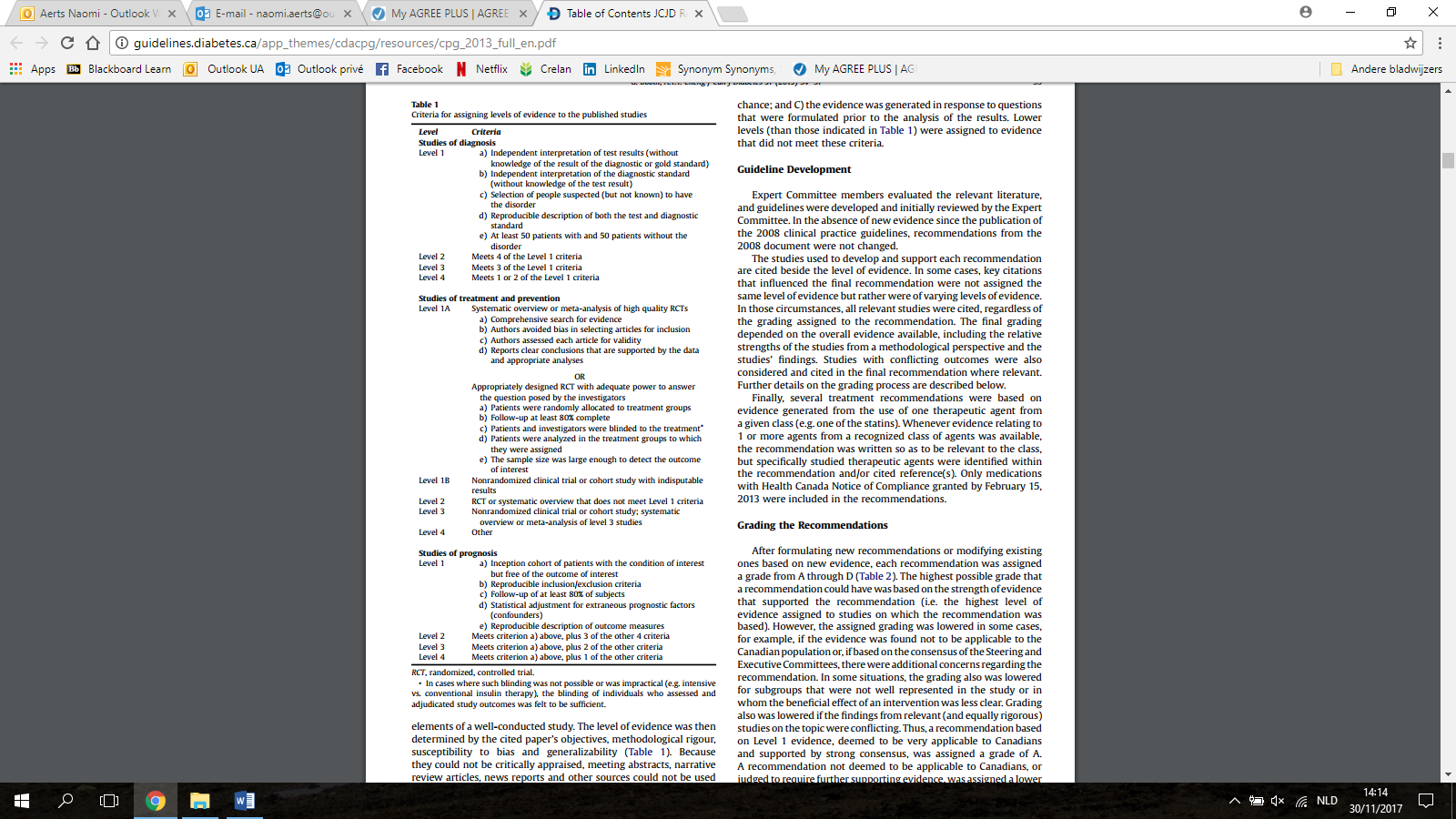


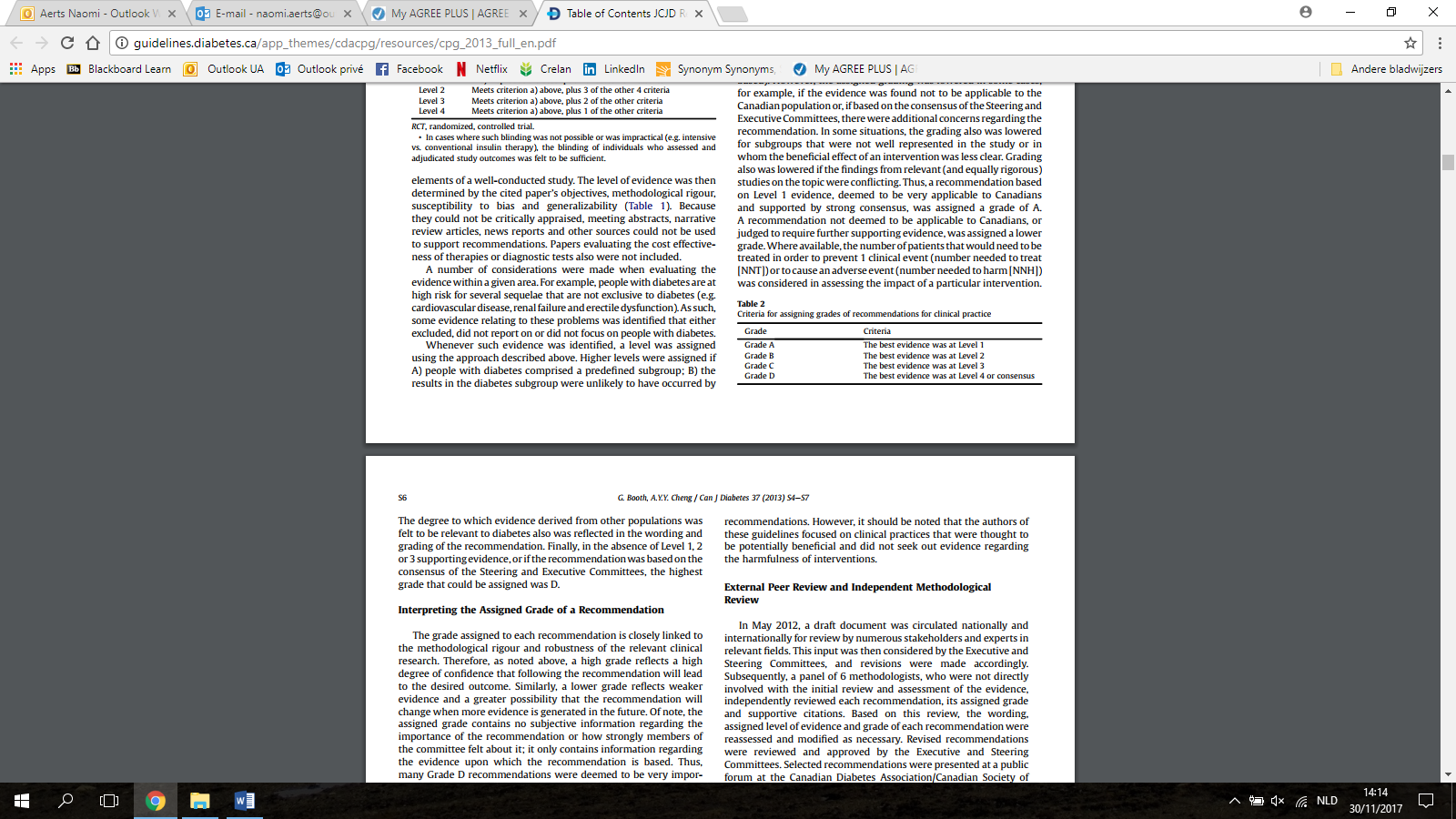


**£££ Definitions**


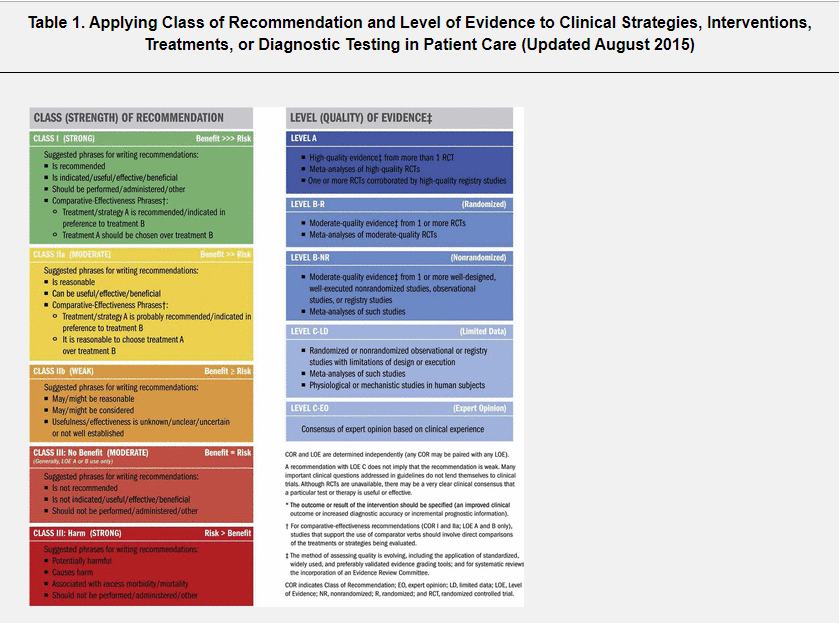

Supplement: Supplementary file 4 — Additional file 4. [file 12875_2021_1409_MOESM4_ESM.zip › Supplementary material 4_GradingR3.docx]
