# Supplementary material for: Systematic review of international clinical guidelines for the promotion of physical activity for the primary prevention of cardiovascular diseases
Source: BMC Fam Pract. 2021 May 19;22:97. doi: 10.1186/s12875-021-01409-9 (PMC8136198; doi:10.1186/s12875-021-01409-9)
Supplement: Supplementary file 4 — Additional file 4. [file 12875_2021_1409_MOESM4_ESM.zip › Supplementary_material_4_GradingR3_UNFIG0001.pdf]

| | |
|---|---|
| UKPDS | United Kingdom Prospective Diabetes Study |
| VADT | Veterans Affairs Diabetes Trial |
| VALUE | Valsartan Antihypertensive Long-Term Use Evaluation |
| VLDL | very low-density lipoprotein |
| $\dot{V}O_2$ | oxygen uptake |
| WHO | World Health Organization |

**Level of evidence**

| | |
|---|---|
| Level of evidence A | Data derived from multiple randomized clinical trials or meta-analyses. |
| Level of evidence B | Data derived from a single randomized clinical trial or large non-randomized studies. |
| Level of evidence C | Consensus of opinion of the experts and/ or small studies, retrospective studies, registries. |

**Classes of recommendations**

| Classes of recommendations | Definition | Suggested wording to use |
|---|---|---|
| Class I | Evidence and/or general agreement that a given treatment or procedure is beneficial, useful, effective. | Is recommended/is indicated |
| Class II | Conflicting evidence and/or a divergence of opinion about the usefulness/efficacy of the given treatment or procedure. | |
| *Class IIa* | *Weight of evidence/opinion is in favour of usefulness/efficacy.* | Should be considered |
| *Class IIb* | *Usefulness/efficacy is less well established by evidence/opinion.* | May be considered |
| Class III | Evidence or general agreement that the given treatment or procedure is not useful/effective, and in some cases may be harmful. | Is not recommended |
