# Supplementary material for: Systematic review of international clinical guidelines for the promotion of physical activity for the primary prevention of cardiovascular diseases
Source: BMC Fam Pract. 2021 May 19;22:97. doi: 10.1186/s12875-021-01409-9 (PMC8136198; doi:10.1186/s12875-021-01409-9)
Supplement: Supplementary file 4 — Additional file 4. [file 12875_2021_1409_MOESM4_ESM.zip › Supplementary_material_4_GradingR3_UNFIG0002.pdf]

SIZE OF TREATMENT EFFECT

ESTIMATE OF CERTAINTY (PRECISION) OF TREATMENT EFFECT

	CLASS I<br>*Benefit >>> Risk*<br>Procedure/Treatment **SHOULD** be performed/administered	CLASS IIa<br>*Benefit >> Risk*<br>*Additional studies with focused objectives needed*<br>**IT IS REASONABLE** to perform procedure/administer treatment	CLASS IIb<br>*Benefit ≥ Risk*<br>*Additional studies with broad objectives needed; additional registry data would be helpful*<br>Procedure/Treatment **MAY BE CONSIDERED**	CLASS III *No Benefit* or CLASS III *Harm*<br>COR III: No benefit — Procedure/Test: Not Helpful; Treatment: No Proven Benefit<br>COR III: Harm — Procedure/Test: Excess Cost w/o Benefit or Harmful; Treatment: Harmful to Patients
**LEVEL A**<br>Multiple populations evaluated*<br>Data derived from multiple randomized clinical trials or meta-analyses	■ Recommendation that procedure or treatment is useful/effective<br>■ Sufficient evidence from multiple randomized trials or meta-analyses	■ Recommendation in favor of treatment or procedure being useful/effective<br>■ Some conflicting evidence from multiple randomized trials or meta-analyses	■ Recommendation's usefulness/efficacy less well established<br>■ Greater conflicting evidence from multiple randomized trials or meta-analyses	■ Recommendation that procedure or treatment is not useful/effective and may be harmful<br>■ Sufficient evidence from multiple randomized trials or meta-analyses
**LEVEL B**<br>Limited populations evaluated*<br>Data derived from a single randomized trial or nonrandomized studies	■ Recommendation that procedure or treatment is useful/effective<br>■ Evidence from single randomized trial or nonrandomized studies	■ Recommendation in favor of treatment or procedure being useful/effective<br>■ Some conflicting evidence from single randomized trial or nonrandomized studies	■ Recommendation's usefulness/efficacy less well established<br>■ Greater conflicting evidence from single randomized trial or nonrandomized studies	■ Recommendation that procedure or treatment is not useful/effective and may be harmful<br>■ Evidence from single randomized trial or nonrandomized studies
**LEVEL C**<br>Very limited populations evaluated*<br>Only consensus opinion of experts, case studies, or standard of care	■ Recommendation that procedure or treatment is useful/effective<br>■ Only expert opinion, case studies, or standard of care	■ Recommendation in favor of treatment or procedure being useful/effective<br>■ Only diverging expert opinion, case studies, or standard of care	■ Recommendation's usefulness/efficacy less well established<br>■ Only diverging expert opinion, case studies, or standard of care	■ Recommendation that procedure or treatment is not useful/effective and may be harmful<br>■ Only expert opinion, case studies, or standard of care
Suggested phrases for writing recommendations	should<br>is recommended<br>is indicated<br>is useful/effective/beneficial	is reasonable<br>can be useful/effective/beneficial<br>is probably recommended or indicated	may/might be considered<br>may/might be reasonable<br>usefulness/effectiveness is unknown/unclear/uncertain or not well established	COR III: No Benefit: is not recommended; is not indicated; should not be performed/administered/other; is not useful/beneficial/effective<br>COR III: Harm: potentially harmful; causes harm; associated with excess morbidity/mortality; should not be performed/administered/other
Comparative effectiveness phrases[†]	treatment/strategy A is recommended/indicated in preference to treatment B<br>treatment A should be chosen over treatment B	treatment/strategy A is probably recommended/indicated in preference to treatment B<br>it is reasonable to choose treatment A over treatment B		
