# Supplementary material for: Systematic review of international clinical guidelines for the promotion of physical activity for the primary prevention of cardiovascular diseases
Source: BMC Fam Pract. 2021 May 19;22:97. doi: 10.1186/s12875-021-01409-9 (PMC8136198; doi:10.1186/s12875-021-01409-9)
Supplement: Supplementary file 4 — Additional file 4. [file 12875_2021_1409_MOESM4_ESM.zip › Supplementary_material_4_GradingR3_UNFIG0003.pdf]

Grading of evidence-based recommendations (EBR)[6]

Grade of recommendation	Description
**A**	Body of evidence can be trusted to guide practice
**B**	Body of evidence can be trusted to guide practice in most situations
**C**	Body of evidence provides some support for recommendation but care should be taken in its application
**D**	Body of evidence is weak and recommendation must be applied with caution

Additional guidance

**CBR**	Consensus-based recommendations: developed by the guidelines expert working group when a systematic review of the evidence found either an absence of direct evidence which answered the clinical question or poor quality evidence, which was deemed not to be strong enough to formulate an evidence-based recommendation.
**PP**	Practice points: developed by the guidelines expert working group where a systematic review had not been conducted but there was a need to provide practical guidance to support the implementation of the evidence-based and/or consensus-based recommendations.
