# Supplementary material for: Systematic review of international clinical guidelines for the promotion of physical activity for the primary prevention of cardiovascular diseases
Source: BMC Fam Pract. 2021 May 19;22:97. doi: 10.1186/s12875-021-01409-9 (PMC8136198; doi:10.1186/s12875-021-01409-9)
Supplement: Supplementary file 4 — Additional file 4. [file 12875_2021_1409_MOESM4_ESM.zip › Supplementary_material_4_GradingR3_UNFIG0004.pdf]

KEY TO EVIDENCE STATEMENTS AND RECOMMENDATIONS	
LEVELS OF EVIDENCE	
1^{++}	High-quality meta-analyses, systematic reviews of RCTs, or RCTs with a very low risk of bias
1^{+}	Well-conducted meta-analyses, systematic reviews, or RCTs with a low risk of bias
1^{-}	Meta-analyses, systematic reviews, or RCTs with a high risk of bias
2^{++}	High-quality systematic reviews of case-control or cohort studies<br>High-quality case-control or cohort studies with a very low risk of confounding or bias and a high probability that the relationship is causal
2^{+}	Well-conducted case-control or cohort studies with a low risk of confounding or bias and a moderate probability that the relationship is causal
2^{-}	Case-control or cohort studies with a high risk of confounding or bias and a significant risk that the relationship is not causal
3	Non-analytic studies, eg case reports, case series
4	Expert opinion
RECOMMENDATIONS	
Some recommendations can be made with more certainty than others. The wording used in the recommendations in this guideline denotes the certainty with which the recommendation is made (the 'strength' of the recommendation).<br>The 'strength' of a recommendation takes into account the quality (level) of the evidence. Although higher-quality evidence is more likely to be associated with strong recommendations than lower-quality evidence, a particular level of quality does not automatically lead to a particular strength of recommendation.<br>Other factors that are taken into account when forming recommendations include: relevance to the NHS in Scotland; applicability of published evidence to the target population; consistency of the body of evidence, and the balance of benefits and harms of the options.	
**R**	For '**strong**' recommendations on interventions that '**should**' be used, the guideline development group is confident that, for the vast majority of people, the intervention (or interventions) will do more good than harm. For '**strong**' recommendations on interventions that '**should not**' be used, the guideline development group is confident that, for the vast **majority** of people, the intervention (or interventions) will do more harm than good.
**R**	For '**conditional**' recommendations on interventions that should be '**considered**', the guideline development group is confident that the intervention will do more good than harm for most patients. The choice of intervention is therefore more likely to vary depending on a person's values and preferences, and so the healthcare professional should spend more time discussing the options with the patient.
GOOD-PRACTICE POINTS	
✓	Recommended best practice based on the clinical experience of the guideline development group.
