# Supplementary material for: Systematic review of international clinical guidelines for the promotion of physical activity for the primary prevention of cardiovascular diseases
Source: BMC Fam Pract. 2021 May 19;22:97. doi: 10.1186/s12875-021-01409-9 (PMC8136198; doi:10.1186/s12875-021-01409-9)
Supplement: Supplementary file 4 — Additional file 4. [file 12875_2021_1409_MOESM4_ESM.zip › Supplementary_material_4_GradingR3_UNFIG0005.pdf]

Table 1
Criteria for assigning levels of evidence to the published studies

Level	*Criteria*
**Studies of diagnosis**	
Level 1	a) Independent interpretation of test results (without knowledge of the result of the diagnostic or gold standard)<br>b) Independent interpretation of the diagnostic standard (without knowledge of the test result)<br>c) Selection of people suspected (but not known) to have the disorder<br>d) Reproducible description of both the test and diagnostic standard<br>e) At least 50 patients with and 50 patients without the disorder
Level 2	Meets 4 of the Level 1 criteria
Level 3	Meets 3 of the Level 1 criteria
Level 4	Meets 1 or 2 of the Level 1 criteria
**Studies of treatment and prevention**	
Level 1A	Systematic overview or meta-analysis of high quality RCTs<br>a) Comprehensive search for evidence<br>b) Authors avoided bias in selecting articles for inclusion<br>c) Authors assessed each article for validity<br>d) Reports clear conclusions that are supported by the data and appropriate analyses<br>OR<br>Appropriately designed RCT with adequate power to answer the question posed by the investigators<br>a) Patients were randomly allocated to treatment groups<br>b) Follow-up at least 80% complete<br>c) Patients and investigators were blinded to the treatment*<br>d) Patients were analyzed in the treatment groups to which they were assigned<br>e) The sample size was large enough to detect the outcome of interest
Level 1B	Nonrandomized clinical trial or cohort study with indisputable results
Level 2	RCT or systematic overview that does not meet Level 1 criteria
Level 3	Nonrandomized clinical trial or cohort study; systematic overview or meta-analysis of level 3 studies
Level 4	Other
**Studies of prognosis**	
Level 1	a) Inception cohort of patients with the condition of interest but free of the outcome of interest<br>b) Reproducible inclusion/exclusion criteria<br>c) Follow-up of at least 80% of subjects<br>d) Statistical adjustment for extraneous prognostic factors (confounders)<br>e) Reproducible description of outcome measures
Level 2	Meets criterion a) above, plus 3 of the other 4 criteria
Level 3	Meets criterion a) above, plus 2 of the other criteria
Level 4	Meets criterion a) above, plus 1 of the other criteria

RCT, randomized, controlled trial.

* In cases where such blinding was not possible or was impractical (e.g. intensive vs. conventional insulin therapy), the blinding of individuals who assessed and adjudicated study outcomes was felt to be sufficient.
