# Supplementary material for: Systematic review of international clinical guidelines for the promotion of physical activity for the primary prevention of cardiovascular diseases
Source: BMC Fam Pract. 2021 May 19;22:97. doi: 10.1186/s12875-021-01409-9 (PMC8136198; doi:10.1186/s12875-021-01409-9)
Supplement: Supplementary file 4 — Additional file 4. [file 12875_2021_1409_MOESM4_ESM.zip › Supplementary_material_4_GradingR3_UNFIG0006.pdf]

Table 2
Criteria for assigning grades of recommendations for clinical practice

Grade	Criteria
Grade A	The best evidence was at Level 1
Grade B	The best evidence was at Level 2
Grade C	The best evidence was at Level 3
Grade D	The best evidence was at Level 4 or consensus
