# Supplementary material for: Systematic review of international clinical guidelines for the promotion of physical activity for the primary prevention of cardiovascular diseases
Source: BMC Fam Pract. 2021 May 19;22:97. doi: 10.1186/s12875-021-01409-9 (PMC8136198; doi:10.1186/s12875-021-01409-9)
Supplement: Supplementary file 4 — Additional file 4. [file 12875_2021_1409_MOESM4_ESM.zip › Supplementary_material_4_GradingR3_UNFIG0007.pdf]

Table 1. Applying Class of Recommendation and Level of Evidence to Clinical Strategies, Interventions, Treatments, or Diagnostic Testing in Patient Care (Updated August 2015)

CLASS (STRENGTH) OF RECOMMENDATION

CLASS I (STRONG) — **Benefit >>> Risk**

Suggested phrases for writing recommendations:
- Is recommended
- Is indicated/useful/effective/beneficial
- Should be performed/administered/other
- Comparative-Effectiveness Phrases†:
  - Treatment/strategy A is recommended/indicated in preference to treatment B
  - Treatment A should be chosen over treatment B

CLASS IIa (MODERATE) — **Benefit >> Risk**

Suggested phrases for writing recommendations:
- Is reasonable
- Can be useful/effective/beneficial
- Comparative-Effectiveness Phrases†:
  - Treatment/strategy A is probably recommended/indicated in preference to treatment B
  - It is reasonable to choose treatment A over treatment B

CLASS IIb (WEAK) — **Benefit ≥ Risk**

Suggested phrases for writing recommendations:
- May/might be reasonable
- May/might be considered
- Usefulness/effectiveness is unknown/unclear/uncertain or not well established

CLASS III: No Benefit (MODERATE) — **Benefit = Risk**
(Generally, LOE A or B use only)

Suggested phrases for writing recommendations:
- Is not recommended
- Is not indicated/useful/effective/beneficial
- Should not be performed/administered/other

CLASS III: Harm (STRONG) — **Risk > Benefit**

Suggested phrases for writing recommendations:
- Potentially harmful
- Causes harm
- Associated with excess morbidity/mortality
- Should not be performed/administered/other

LEVEL (QUALITY) OF EVIDENCE‡

LEVEL A
- High-quality evidence‡ from more than 1 RCT
- Meta-analyses of high-quality RCTs
- One or more RCTs corroborated by high-quality registry studies

LEVEL B-R — **(Randomized)**
- Moderate-quality evidence‡ from 1 or more RCTs
- Meta-analyses of moderate-quality RCTs

LEVEL B-NR — **(Nonrandomized)**
- Moderate-quality evidence‡ from 1 or more well-designed, well-executed nonrandomized studies, observational studies, or registry studies
- Meta-analyses of such studies

LEVEL C-LD — **(Limited Data)**
- Randomized or nonrandomized observational or registry studies with limitations of design or execution
- Meta-analyses of such studies
- Physiological or mechanistic studies in human subjects

LEVEL C-EO — **(Expert Opinion)**

Consensus of expert opinion based on clinical experience

COR and LOE are determined independently (any COR may be paired with any LOE).

A recommendation with LOE C does not imply that the recommendation is weak. Many important clinical questions addressed in guidelines do not lend themselves to clinical trials. Although RCTs are unavailable, there may be a very clear clinical consensus that a particular test or therapy is useful or effective.

* The outcome or result of the intervention should be specified (an improved clinical outcome or increased diagnostic accuracy or incremental prognostic information).

† For comparative-effectiveness recommendations (COR I and IIa; LOE A and B only), studies that support the use of comparator verbs should involve direct comparisons of the treatments or strategies being evaluated.

‡ The method of assessing quality is evolving, including the application of standardized, widely used, and preferably validated evidence grading tools; and for systematic reviews the incorporation of an Evidence Review Committee.

COR indicates Class of Recommendation; EO, expert opinion; LD, limited data; LOE, Level of Evidence; NR, nonrandomized; R, randomized; and RCT, randomized controlled trial.
