# Supplementary material for: Systematic review of international clinical guidelines for the promotion of physical activity for the primary prevention of cardiovascular diseases
Source: BMC Fam Pract. 2021 May 19;22:97. doi: 10.1186/s12875-021-01409-9 (PMC8136198; doi:10.1186/s12875-021-01409-9)
Supplement: Supplementary file 5 — Additional file 5. [file 12875_2021_1409_MOESM5_ESM.docx]

**APPENDIX 1 -** AGREE Scores: Overview AGREE scores per domain of included CPGs

| Guideline code, year (ref.) | Domain 1 Scope and Purpose (%) | Domain 2 Stakeholder Involvement (%) | Domain 3 Rigour of Development (%) | Domain 4 Clarity of Presentation (%) | Domain 5  Applicability (%) | Domain 6  Editorial Independence (%) | Overall Score (%) |
| --- | --- | --- | --- | --- | --- | --- | --- |
| CVD 1, 2012 (1) | 91 | 87 | 81 | 85 | 78 | 78 | 89 |
| CVD 2, 2014 (2) | 86 | 61 | 82 | 94 | 54 | 79 | 83 |
| CVD 3, 2016 (3) | 81 | 44 | 49 | 86 | 48 | 46 | 58 |
| CVD 4, 2017 (4) | 89 | 58 | 57 | 72 | 50 | 58 | 83 |
| CVD 5, 2019 (5) | 75 | 89 | 79 | 92 | 67 | 88 | 83 |
| CVD 6, 2018 (6) | 67 | 47 | 67 | 83 | 69 | 83 | 75 |
| CVD 7, 2019 (7) | 69 | 61 | 74 | 86 | 46 | 75 | 75 |
| LSt 1, 2012  (8) | 89 | 72 | 77 | 64 | 52 | 83 | 75 |
| LSt 2, 2014  (9) | 58 | 53 | 74 | 81 | 25 | 75 | 83 |
| LSt 3, 2014  (10) | 72 | 67 | 72 | 64 | 69 | 42 | 75 |
| OW 1, 2012 (11) | 53 | 36 | 74 | 72 | 65 | 79 | 67 |
| OW 2, 2013 (12) | 86 | 81 | 77 | 78 | 71 | 67 | 83 |
| OW 3, 2014 (13) | 58 | 44 | 68 | 81 | 29 | 54 | 75 |
| OW 4, 2014 (14) | 97 | 69 | 71 | 92 | 50 | 10 | 75 |
| OW 5, 2014 (15) | 75 | 69 | 71 | 81 | 75 | 83 | 75 |
| OW 6, 2015 (16) | 81 | 44 | 71 | 78 | 71 | 75 | 75 |
| OW 7, 2015 (17) | 64 | 31 | 65 | 56 | 42 | 46 | 75 |
| LCh 1, 2014 (18) | 89 | 67 | 77 | 58 | 71 | 25 | 75 |
| LCh 2, 2014 (19) | 75 | 53 | 71 | 86 | 58 | 58 | 92 |
| LCh 3, 2018 (20) | 81 | 72 | 82 | 86 | 67 | 79 | 83 |
| LCh 4, 2019 (21) | 39 | 47 | 65 | 83 | 52 | 67 | 75 |
| BP 1, 2014 (22) | 78 | 53 | 71 | 94 | 58 | 29 | 75 |
| BP 2, 2014 (23) | 64 | 47 | 63 | 67 | 65 | 42 | 67 |
| BP 3, 2020 (24) | 75 | 58 | 77 | 86 | 69 | 75 | 75 |
| DM 1, 2013 (25) | 72 | 69 | 83 | 81 | 54 | 67 | 75 |
| DM 2, 2014 (26) | 86 | 67 | 66 | 67 | 56 | 10 | 67 |
| DM 3, 2019 (27) | 58 | 36 | 57 | 89 | 54 | 83 | 75 |

1. National Vascular Disease Prevention Alliance. Guidelines for the management of absolute CVD risk [Guideline]. 2012 [Available from: <https://www.strokefoundation.com.au/~/media/strokewebsite/resources/treatment/absolutecvd_gl_webready.ashx?la=en>.

2. NICE - National Institute for Health + Care Excellence. Prevention of cardiovascular disease (PH25) [Guideline]. 2010 [updated 31.12.2015. Available from: <http://guidance.nice.org.uk/PH25>.

3. Piepoli MF, Hoes AW, Agewall S, Albus C, Brotons C, Catapano AL, et al. 2016 European Guidelines on cardiovascular disease prevention in clinical practiceThe Sixth Joint Task Force of the European Society of Cardiology and Other Societies on Cardiovascular Disease Prevention in Clinical Practice (constituted by representatives of 10 societies and by invited experts)Developed with the special contribution of the European Association for Cardiovascular Prevention &amp; Rehabilitation (EACPR). European heart journal. 2016;37(29):2315-81.

4. SIGN - Scottish Intercollegiate Guidelines Network. Risk estimation and the prevention of cardiovascular disease (SIGN CPG 149) [Guideline]. 2017 [Available from: <http://www.sign.ac.uk/sign-149-risk-estimation-and-the-prevention-of-cardiovascular-disease.html>.

5. Practitioners N-DCoG. Cardiovascular risk management (M84) Netherlands: NHG; 2019 [updated June 2019. Version 4.0:[Available from: <https://richtlijnen.nhg.org/standaarden/cardiovasculair-risicomanagement#volledige-tekst-literatuur>.

6. Atherton JJ, Sindone A, De Pasquale CG, Driscoll A, MacDonald PS, Hopper I, et al. National Heart Foundation of Australia and Cardiac Society of Australia and New Zealand: Guidelines for the Prevention, Detection, and Management of Heart Failure in Australia 2018. Heart, lung & circulation. 2018;27(10):1123-208.

7. Arnett DK, Blumenthal RS, Albert MA, Buroker AB, Goldberger ZD, Hahn EJ, et al. 2019 ACC/AHA Guideline on the Primary Prevention of Cardiovascular Disease: A Report of the American College of Cardiology/American Heart Association Task Force on Clinical Practice Guidelines. Circulation. 2019;140(11):e596-e646.

8. National Guideline C. Behavioral counseling interventions to promote a healthful diet and physical activity for cardiovascular disease prevention in adults: U.S. Preventive Services Task Force recommendation statement. 2012.

9. National Guideline C. 2013 AHA/ACC guideline on lifestyle management to reduce cardiovascular risk: a report of the American College of Cardiology/American Heart Association Task Force on Practice Guidelines. 2014.

10. NICE - National Institute for Health + Care Excellence. Behaviour change: individual approaches (PH49) [Guideline]. 2014 [Available from: <http://guidance.nice.org.uk/PH49>.

11. National Guideline C. Screening for and management of obesity in adults: U.S. Preventive Services Task Force recommendation statement. 2012.

12. National Guideline C. Clinical practice guidelines for the management of overweight and obesity in adults, adolescents and children in Australia. 2013.

13. National Guideline C. 2013 AHA/ACC/TOS guideline for the management of overweight and obesity in adults: a report of the American College of Cardiology/American Heart Association Task Force on Practice Guidelines and The Obesity Society. 2014.

14. National Guideline C. VA/DoD clinical practice guideline for screening and management of overweight and obesity. 2014.

15. NICE - National Institute for Health + Care Excellence. Obesity (CG43) [Guideline]. 2014 [Available from: <http://guidance.nice.org.uk/CG43>.

16. National Guideline C. Recommendations for prevention of weight gain and use of behavioural and pharmacological interventions to manage overweight and obesity in adults in primary care. 2015.

17. National Guideline C. Maintaining a healthy weight and preventing excess weight gain among adults and children. 2015.

18. NICE - National Institute for Health + Care Excellence. Cardiovascular disease: risk assessment and reduction, including lipid modifiation (cg181) [Guideline]. 2014 [Available from: <http://guidance.nice.org.uk/CG67>.

19. National Guideline C. VA/DoD clinical practice guideline for the management of dyslipidemia for cardiovascular risk reduction. 2014.

20. Grundy SM, Stone NJ, Bailey AL, Beam C, Birtcher KK, Blumenthal RS, et al. 2018 AHA/ACC/AACVPR/AAPA/ABC/ACPM/ADA/AGS/APhA/ASPC/NLA/PCNA Guideline on the Management of Blood Cholesterol: A Report of the American College of Cardiology/American Heart Association Task Force on Clinical Practice Guidelines. Circulation. 2019;139(25):e1082-e143.

21. Mach F, Baigent C, Catapano AL, Koskinas KC, Casula M, Badimon L, et al. 2019 ESC/EAS Guidelines for the management of dyslipidaemias: lipid modification to reduce cardiovascular risk: The Task Force for the management of dyslipidaemias of the European Society of Cardiology (ESC) and European Atherosclerosis Society (EAS). European heart journal. 2019;41(1):111-88.

22. National Guideline C. VA/DoD clinical practice guideline for the diagnosis and management of hypertension in the primary care setting. 2014.

23. National Guideline C. Team-based care to improve blood pressure control: recommendation of the Community Preventive Services Task Force. 2014.

24. Rabi DM, McBrien KA, Sapir-Pichhadze R, Nakhla M, Ahmed SB, Dumanski SM, et al. Hypertension Canada's 2020 Comprehensive Guidelines for the Prevention, Diagnosis, Risk Assessment, and Treatment of Hypertension in Adults and Children. The Canadian journal of cardiology. 2020;36(5):596-624.

25. Cheng AY. Canadian Diabetes Association 2013 clinical practice guidelines for the prevention and management of diabetes in Canada. Introduction. Can J Diabetes. 2013;37 Suppl 1:S1-3.

26. NICE - National Institute for Health + Care Excellence. Preventing type 2 diabetes - population and community interventions (PH35) [Guideline]. 2011 [Available from: <http://guidance.nice.org.uk/PH35>.

27. Cosentino F, Grant PJ, Aboyans V, Bailey CJ, Ceriello A, Delgado V, et al. 2019 ESC Guidelines on diabetes, pre-diabetes, and cardiovascular diseases developed in collaboration with the EASD: The Task Force for diabetes, pre-diabetes, and cardiovascular diseases of the European Society of Cardiology (ESC) and the European Association for the Study of Diabetes (EASD). European heart journal. 2019;41(2):255-323.
